# Tripartite evolutionary game and simulation analysis of agricultural non-point source pollution control

**DOI:** 10.1371/journal.pone.0305191

**Published:** 2024-06-28

**Authors:** Zhilin Wang, Hangbiao Shang

**Affiliations:** College of Economics and Management, Northeast Forestry University, Harbin, P.R. China; Khwaja Fareed University of Engineering & Information Technology, PAKISTAN

## Abstract

Agricultural non-point source pollution control (ANSPC) is a complex, long-term and dynamic environmental protection process. In order to motivate multiple subjects to participate in ANSPC, this paper constructs a tripartite evolutionary game model of local government, village collectives and farmers, which explores the strategic choices and influencing factors of different subjects through simulation analysis. The results indicate that: There are five stable strategy points in the ANSPC game system, which can be divided into four stages based on subject interactions. Village collectives should play an intermediary role in ANSPC and try to coordinate the behaviour of different subjects. The ideal and stable evolution state is "weak supervise, positive response, and active participate", but it cannot be realized at present. The strategy selection of subjects is determined by relative net income. Providing penalties requires considering the heterogeneity of subjects, but incentives are beneficial for achieving tripartite governance. This study provides new evidence for understanding the role of multi-agency participation in agricultural non-point source pollution control, and provides theoretical guidance for the government to formulate differentiated intervention mechanisms, which is an important reference for achieving sustainable development goals.

## Introduction

Agricultural ecosystems are crucial for human survival and development, combining economic, social and ecological values [1]. However, owing to the long-term adoption of the "high input, high output" production model, the overuse of chemicals has led to serious non-point source pollution [2]. Recent studies have demonstrated that over 64% of farmland worldwide is exposed to non-point source pollution, especially in agriculture-based developing countries [3]. Agricultural non-point source pollution has become one of the important factors constraining the high-quality development and protection of farmland ecosystems [4]. Against this background, it is worthwhile to investigate the optimal path of agricultural non-point source pollution control (ANSPC) through system design.

In fact, many countries, including China, aim to reduce the potential for non-point source pollution at the source through grassroots system building, i.e., encouraging multiple stakeholder participation [5]. For example, the State Council issued the Program for Cultivating and Developing Agricultural Non-point Source Pollution Control and Rural Sewage and Waste Treatment Market Entities in 2016, highlighting the formation of a government-led and farmer-participatory mechanism. Furthermore, the Ministry of Agriculture and Rural Development issued the Implementation Plan for Agricultural Non-point Source Pollution Control and Supervision and Guidance (for Trial Implementation) in 2021, placing more emphasis on the role of village collectives on the above basis. It is undeniable that ANSPC in China has achieved gradual success: from 2015 to 2020, the use of fertilisers and pesticides decreased by 13% and 14%, respectively, and both utilisation rates exceeded 40% [6]. However, agricultural non-point source pollution problem has not been completely solved [7]. There are various problems in practice. For example, the implementation of rural policies is inadequate [8], the role of village collectives is misplaced [9] and the willingness of farmers to participate is high, but the level of participation is low [10]. Extensive evidence demonstrates that village collectives have insufficient awareness of the importance of ANSPC policy [11]. As mentioned by Tian and Tsai (2023), the incentive and penalty mechanisms of ANSPC in China are not complete, and the subjective status of village collectives is not clear, which consequently prevents farmers from being promptly embedded in the control process [12]. What’s more, although the government spent considerable sums annually on control, the grassroots system, due to the lack of coordination with village collectives, had not brought positive feedback, but increased the government’s financial burden instead [13]. Therefore, it is necessary to investigate the strategic choices and stabilizing conditions of different subjects and drivers to improve the efficiency of ANSPC and build a long-term regulatory mechanism.

Although scholars have conducted a series of studies on government-cooperative [14] and government-farmer [15] interactions, they had not explained the dynamic formation process of the grassroots system in ANSPC. Most studies consider the government and farmers as key subjects. Limited by information asymmetry and transaction costs, the farmers’ endogenous motivation to participate in ANSPC is insufficient, resulting in opportunistic behaviour [16]. In addition, local governments may adopt different strategies due to the different costs and benefits of monitoring, leading to the failure of macro policies directly to micro farmers. Existing studies suggest that village collectives can often be seen as dual agents of the government and farmers and act as a link between them [17]. Village collectives should coordinate the interests and demands of all parties and be embedded in ANSPC [18]. Therefore, it is important to consider the role of village collectives in ANSPC. However, the question of how local governments, village collectives, and farmers interact with each other to influence ANSPC has not been noticed and answered in previous literature.

To fill these gaps mentioned above, this paper constructs a tripartite evolutionary game model of local government, village collectives and farmers. In addition, based on the context of ANSPC in China, we established a realistic database to examine the effects of key parameter changes on subjects’ strategy choices. In this study, the evolutionary game model is an appropriate tool for the following reasons: First, evolutionary game theory argues a continuous change in subjects’ strategy choices. This can explain the phenomenon of subjects learning and competing with others [19]. Second, evolutionary game theory assumes that subjects are boundedly rational [20]. Obviously, ANSPC is a complex, dynamic and repeated bargaining process, and each subject’s strategy choice is the result of comprehensive consideration of costs and benefits. Therefore, the evolutionary game model is suitable for the practice of ANSPC.

The possible innovations of this study are summarized below. First, few studies have been conducted on the role of village collectives in agricultural non-point source pollution control, and most of them are case studies. This paper analyses the role of village collectives in ANSPC based on the factors of benefits, subsidies, costs and losses, and gives evolutionary strategies for different contexts, which enriches the theoretical framework of ANSPC. Second, this study provides a new perspective on the implementation of ANSPC. Existing studies mainly focus on the causes, influencing factors and behaviour with ANSPC, while the study of subject interaction is neglected. Based on the subject being boundedly rational, we construct a unified framework of the game among local government, village collectives and farmers, which dynamically demonstrates the process of the subject’s strategy choice. This study contributes to exploring the optimal path of ANSPC and also provides management ideas for other countries facing the same problem. Third, this study identifies the influencing factors that affect the subject’s strategy choice in ANSPC. We theoretically demonstrate that incentives and penalties have different effects on the willingness of different subjects, which contributes to formulate differentiated intervention mechanisms by the government.

## Literature review

### Literature on agricultural non-point source pollution and control

Agricultural non-point source pollution is the pollution of soil, water and air by nutrients due to the irrational disposal of agricultural chemicals and wastes, driven by precipitation and topography [21]. Existing literatures mainly focus on two aspects: influencing factors and treatment technologies. First, the influencing factors can be further divided into individual and social components. On the one hand, the theory of planned behaviour argues that individual perceptions and willingness are important factors influencing agricultural non-point source pollution [22]. Agricultural non-point source pollution has been studied in terms of the fertilizer use, straw burning, and cropping structure [23]. In addition, Blackstock (2010) argued that social networks and social capital can reduce agricultural non-point source pollution levels [24]. On the other hand, the social part investigated the relationship between factors such as economic level [25], urbanization [26], environmental regulation [27] and industrial transformation [28] with non-point source pollution.

The second group of related literature is on treatment technology. Overall, considering the subject heterogeneity, the treatment technologies include structural and non-structural measures. Structural measures are government-driven, which utilize ecological engineering to reduce the hazards of pollutants. For example, Kumwimba et al. (2018) demonstrated that vegetated drainage ditches technology has great potential to reduce agricultural runoff and domestic wastewater [29]. Other measures include the creation of constructed wetland, artificial floating island and riparian vegetation buffer zone [30]. Non-structural measures put more emphasis on the participation of micro-individuals to reduce pollutant emissions at the source. As mentioned in the introduction, agricultural non-point source pollution is usually caused by inappropriate environmental behaviours. Therefore, previous studies have attempted to change the production behaviour of farmers from different perspectives. For example, Okmah (2018) explored the relationship between "awareness-behaviour-water quality" and indicated that increased awareness of pesticide management contributed to farmers’ behavioural changes and reduced fertilizer use, which in turn improved water quality [31].

The above literatures systematically discuss the macro-mechanisms of ANSPC and provide the necessary theoretical references for this study. However, most studies have only considered the individual control behaviours of the government and farmers, without considering the role played by village collectives. Meanwhile, they analyse the problem from a static perspective without emphasizing the long-term interaction between the subjects.

### Literature on subject participation in ANSPC

ANSPC must rely on the government’s financial security power and administrative enforcement power. First, a fiscal transfer is the main funding source for ANSPC, compensating for the dilemma of underinvestment in rural resources under the urban–rural dual structure [32]. Huber et al. (2023) conducted modelling based on subject behaviour and simulated the impact of different subsidies on farmers’ perceived costs and benefits in Switzerland, suggesting that subsidies are the key factor in agricultural technology adoption [33]. Based on constructing a triple game, Tian et al. (2022) argued that government subsidies are the major influential factor in promoting fertiliser reduction and expanding green consumption [15]. Second, due to the public nature of the environment, the legal responsibility of government regulation is reasonable, necessary and urgent [34]. Bildirici (2022) analysed the impact of governance on environmental pollution with panel data for a number of countries in the Middle East and Sub-Saharan Africa, and found that lack of governance resulted in continued environmental degradation [35]. The above research provides a theoretical basis for the government to get directly involved in ANSPC; however, different government levels have different roles [36]. Sheng and Webber (2017) concluded that the authority of environmental governance in China is decentralised, with the central government providing funds, while the local government is in charge of fund allocation and regulation [37]. The scope of this research is the end of ANSPC and does not include providing funds. Based on the above analysis, the definition of the subject in this study is the local government.

Although the government has designed the ANSPC system, the downstream policy must be improved by village collectives, which in China include village committees, new agricultural organisations and new social organisations [38]. Traditionally, village collectives are required to actively implement government policies on ANSPC and to perform the function of transferring information from top to bottom [39]. Poudyal et al. (2023) concluded that community collective action played a central role in Nepal’s forest transformation, which required a re-conceptualization of the policy implementation model [40]. Unfortunately, village collectives may have become independent economic entities due to the lack of incentive mechanisms for farmer cadres [41]. In addition, the lack of trust between subjects makes it easy to generate rent-seeking behaviour. Scholars generally believe that the lack of a mechanism to constrain village collectives would cause a crowding-out effect on the benefits to the government and farmers [42].

Finally, farmers are both the perpetrators and beneficiaries of agricultural non-point source pollution and their level of participation is a critical issue that must be addressed urgently. In terms of passivity, weak environmental awareness and lack of participatory power are the factors that lead to farmers’ negative participation [43]. Conversely, external factors, such as policy fragmentation, one-off projects and poor communication, also limit their ability to participate in ANSPC [44]. Regarding initiatives, researchers have found that farmers’ benefits are fundamental to sustaining rural projects. Furthermore, there are also studies on the outcomes of farmers’ participation. Feng et al. (2022) examined the path of farmers’ role in water management and concluded that farmers’ participation improves social cohesion and can play a monitoring role [17].

Above literatures have investigated the necessity of different subjects’ participation in ANSPC and the game relationship involved. However, there is still insufficient discussion in the field of ANSPC grassroots system building, especially the construction of the game model of "local government—village collectives—farmers".

## Game subjects and behaviour analysis

### Analysis of local governments’ behaviour

Local governments play the dual role of implementer and regulator in the ANSPC system. First, rural environmental construction is essential to local government performance evaluation. The government needs to be at the centre because of the low participation of village collectives and farmers due to ANSPC externalities. Second, rural areas require different levels of support. Local governments may waste funds if they adopt a uniform allocation, whereas adequate research can increase implementation costs. Finally, local governments should regulate the behaviour of other subjects. With strong regulation, local governments can punish negative subjects and receive positive influence from the performance evaluation. In the case of weak regulation, the local government can be negatively influenced by the farmers’ reports.

### Analysis of village collectives’ behaviour

Village collectives play a bridging role in the ANSPC process. As a self-governing organisation of farmers, village collectives are more widely and directly connected to the farmers. Furthermore, few farmers can directly understand ANSPC policies, so the demonstration and guidance role of village collectives must be emphasised [17]. Village collectives have two strategic options: positive response and negative response. The former can play a proper guiding role by creating the atmosphere of ANSPC and defending the interests of the farmers. Due to the project’s impact on the farmers, the actions of the village collectives are extensive and costly; therefore, they may choose a negative response.

### Analysis of farmers’ behaviour

The farmers were the participants and beneficiaries. Farmers have two choices of ‘active participation and passive participation’ in ANSPC, which are constrained by various factors. On the one hand, ANSPC can improve the farmers’ quality of life and substantially benefit them. Farmers with a strong sense of identity actively participate in ANSPC and report negative issues to protect their interests. In contrast, farmers in the start-up phase pay more attention to their interests. Individually, they show a weak awareness of environmental protection and serious dependence on village collectives. In addition, ANSPC can increase the cost of living due to the requirement of agricultural film recycling and pesticide reduction; thus, farmers may be biased towards passive participation.

## Materials and methods

### Basic assumptions

This paper proposes the following hypothesis based on the above analysis, and the specific descriptions of parameters are shown in Table 1.

**Table 1:** Parameter setting.

Subject	Parametric	Definition
Local government	*R*_*g*_	Daily benefit
	*B*_*g*_	Additional benefits
	*A*	Subsidy provided by the central government
	*T*_*g*1_/*T*_*g*2_	Subsidy to village collective
	*C*_*g*_/Δ*G*	Cost by weak/strong regulation
	*αD*_*g*_	Additional loss
Village collective	*B*_*v*1_	Environment benefit
	*B*_*v*2_	Reputation effect
	*M*_*p*1_	Reward to farmer
	*C*_*p*_/Δ*V*	Cost by negative/positive regulation
	*F*_*v*_	Fine imposed by the local government
	*D*_*v*1_	Environmental loss
	*αD*_*v*2_	Additional loss
	*αF*_*v*_	Penalty
Farmer	*U*_1_/*U*_2_	Farmers’ benefit
	*T*_*p*1_	Reward by local government
	*T*_*p*2_	Compensation by village collective
	*C*_*p*1_	Cost
	*C*_*p*2_	Report cost
	*α*	The probability of farmers reporting and being successful
	*β*	The probability that farmers damage the environment
	*βF*_*p*_	Fine for farmers

Hypothesis 1: Subjects. The game has three subjects—local government, village collectives and farmers—all of whom are finitely rational, want to achieve the final goal at the minimum cost and must adjust several times when the information is asymmetric. With cost-benefit measurements, they eventually converge to stability.

Hypothesis 2: Strategies. The behavioural strategies of local government are ‘strong or weak regulation’; the probability of choosing the former is *x*, while the latter is 1−*x*.The behavioural strategies of village collectives are ‘positive or negative response’; the probability of choosing the former is *y*, while the latter is 1−*y*.The behavioural strategies of farmers are ‘active or passive participation’; the probability of choosing the former is *z*, while the latter is 1−*z*.

Hypothesis 3: Benefits. *R*_*g*_ is the daily benefit of the local government, while *R*_*v*_ is the daily benefit of grassroots institutions. *B*_*v*1_ is the benefit to the environment (e.g. population quality improvement, infrastructure construction, poverty alleviation, etc.) when village collectives respond positively. *U*_1_ is the farmers’ benefit if the village collectives respond positively. *U*_2_ is the farmers’ benefit if they respond negatively (*U*_1_>*U*_2_).*B*_*g*_ are the additional benefits, such as credibility and performance evaluation, brought by the discovery of strong local government regulation when farmers actively participate. *B*_*v*2_ is the reputation effect brought by the discovery of the positive response of village collectives.

Hypothesis 4: Subsidies. *A* is the subsidy provided by the central government to encourage the local government to choose a strong regulatory strategy. *T*_*g*1_ is the subsidy given by the local government when it chooses a strong regulation from the actual situation. *T*_*g*2_ is the subsidy given at a uniform standard when the local government chooses a weak regulation strategy, and *T*_*g*1_≥*T*_*g*2_.*M*_*p*1_ is the reward given to farmers when village collectives respond positively, farmers actively participate, and *B*_*v*1_>*M*_*p*1_.*T*_*p*1_ is the reward given to farmers by the local government when they report that village collectives respond negatively. *T*_*p*2_ is the compensation given to the farmers by the village collectives when the farmers report the negative response of the village collectives.

Hypothesis 5: Costs. *C*_*g*_ is the cost when the local government adopts weak regulation (issuing policy orders, etc.). Δ*G* is the incremental cost when the local government opts for strong regulation (conducting field regulation, etc.). *C*_*p*_ is the cost if the village collectives choose a negative response (failing to educate, controlling only at critical times, etc.). Δ*V* is the incremental cost when village collectives choose a positive response (various forms of education, suitable regulatory mechanisms, cooperation with local governments, etc.). *C*_*p*1_ is the cost of farmers’ participation, while *C*_*p*2_ is the cost of reporting by the farmers. *α* is the probability of farmers reporting and being successful, and 0<*α*<1.

Hypothesis 6: Losses. *F*_*v*_ is the fine imposed by the local government if the village collectives react negatively. *D*_*v*1_ is the environmental loss when the village collectives respond negatively. *αD*_*g*_ is the additional loss of weak local government regulation when farmers actively participate. *αD*_*v*2_ is the additional loss of reputation when village collectives respond negatively. *αF*_*v*_ is the penalty for village collectives being reported when local governments are weakly regulated. *β* is the probability that farmers participate passively and damage the environment. *βF*_*p*_ is the fine for farmers who damage the environment when the local government supervises strongly, or the village collectives responds positively.

### Model construction

Table 2 shows the construction of the tripartite game matrix of local government, village collectives and farmers.

**Table 2:** Triple game payoff matrix.

Local government	Village collective	Farmer	
		Active participation (*z*)	Passive participation (1−*z*)
Strong regulation (*x*)	Positive response (*y*)	*R*_*g*_−*C*_*g*_−Δ*G*−*T*_*g*1_+*A*+*B*_*g*_, *R*_*v*_−*C*_*v*_−Δ*V*+*B*_*v*1_−*M*_*p1*_+*B*_*v*2_, *U*_1_−*C*_*p*1_+*M*_*p*1_	*R*_*g*_−*C*_*g*_−Δ*G*−*T*_*g*1_+*A*+*βF*_*p*_, *R*_*v*_−*C*_*v*_−Δ*V*+*B*_*v*1_, *U*_1_−*βF*_*p*_
	Negative response (1−*y*)	*R*_*g*_−*C*_*g*_−Δ*G*−*T*_*g*1_+*F*_*v*_+*A*−*αT*_*p*1_+*B*_*g*_, *R*_*v*_−*C*_*v*_−*D*_*v*1_−*αT*_*p*2_−*F*_*v*_−α_*v*2_, *U*_2_−*C*_*p*1_−*C*_*p*2_+*αT*_*p*1_+*αT*_*p*2_	*R*_*g*_−*C*_*g*_−Δ*G*−*T*_*g*1_+*F*_*v*_*+A*+*βF*_*p*_, *R*_*v*_−*C*_*v*_−*D*_*v*1_−*F*_*v*_, *U*_2_−*βF*_*p*_
Weak regulation (1−*x*)	Positive response (*y*)	*R*_*g*_−*C*_*g*_−*T*_*g*2_−*αD*_*g*_, *R*_*v*_−*C*_*v*_−Δ*V*+*B*_*v*1_−*M*_*p1*_+*B*_*v*2_, *U*_1_−*C*_*p*1_+*M*_*p*1_	*R*_*g*_−*C*_*g*_−*T*_*g*2_, *R*_*v*_−*C*_*v*_−Δ*V*+*B*_*v*1_, *U*_1_
	Negative response (1−*y*)	*R*_*g*_−*C*_*g*_−*T*_*g*2_+α*F*_*v*_−*αT*_*p*1_−*αD*_*g*_, *R*_*v*_−*C*_*v*_−*D*_*v*1_−*αT*_*p2*_−*αF*_*v*_−*αD*_*v*2_, *U*_2_−*C*_*p*1_−*C*_*p*2_+*αT*_*p*1_+*αT*_*p*2_	*R*_*g*_−*C*_*g*_−*T*_*g*2_, *R*_*v*_−*C*_*v*_−*D*_*v1*_, *U*_2_

## Equilibrium strategy analysis

### Single subject

#### Local government regulatory behaviour

The expected utilities of strong and weak local government regulations are *U*_1*x*_,*U*_2*x*_ and the average expected utilities are $\bar{U_{x}}$. The expressions are:

$$U_{1x}=yz(R_{g}-C_{g}-\Delta G-T_{g1}+A+B_{g})+y(1-z)(R_{g}-C_{g}-\Delta G-T_{g1}+A+\beta F_{p})+(1-y)z(R_{g}-C_{g}-\Delta G-T_{g1}+F_{v}+A-{\alpha T}_{p1}+B_{g})+(1-y)(1-z)(R_{g}-C_{g}-\Delta G-T_{g1}+F_{v}+A+\beta F_{p})$$ (1)

$$U_{2x}=yz(R_{g}-C_{g}-T_{g2}-\alpha D_{g})+y(1-z)(R_{g}-C_{g}-T_{g2})+(1-y)z(R_{g}-C_{g}-T_{g2}+\alpha F_{v}-{\alpha T}_{p1}-\alpha D_{g})$$ (2)

$$\bar{U_{x}}=xU_{1x}+(1-x)U_{2x}$$ (3)

The replicated dynamic equation *F*(*X*) for the local government choosing a robust regulatory strategy is as follows:

$$F(X)=\frac{dx}{dt}=x(U_{1x}-\bar{U_{x}})=x(1-x)[\left(T_{g2}-T_{g1}-\Delta G+F_{v}+A+\beta F_{p})+yz\alpha F_{v}-yF_{v}+z(B_{g}+\alpha D_{g}-\alpha F_{v}-\beta F_{p}\right)]$$ (4)

Let *F*(*X*) = 0 and note $z^{*}=\frac{(T_{g1}+\Delta G-T_{g2}-F_{v}-A-\beta F_{p})+yF_{v}}{y\alpha F_{v}+(B_{g}+\alpha D_{g}-\alpha F_{v}-\beta F_{p})}$; then, the evolutionary stability point of local government regulation is analysed as follows:

(1) If *z* = z*, then the replicated dynamic equation *F*(*X*) ≡ 0 and all regulatory decisions *x* are evolutionary equilibrium points. Regardless of the initial proportion of local governments choosing ‘strong regulation’ or ‘weak regulation’, the outcome does not change over time.
(2) If *z*≠z*. Let *F*(*X*) = 0, then *x* = 0 and *x* = 1 are two possible equilibrium points. From the stability condition of the replicated dynamic equation, it can be deduced that if $\frac{dX}{dx}=(1-2x)[\left(T_{g2}-T_{g1}-\Delta G+F_{v}+A+\beta F_{p})+yz\alpha F_{v}-yF_{v}+z(B_{g}+\alpha D_{g}-\alpha F_{v}-\beta F_{p}\right)]<0$, the result is the stable point of the evolutionary game. In particular, two different cases were discussed:
1) When $0<z<z^{*},{\frac{dF(X)}{dx}|}_{x=0}<0$ and ${\frac{dF(X)}{dx}|}_{x=1}>0.x=0$ is the equilibrium point of the evolutionary game, and the final result is to choose a weak regulatory strategy.
2) When $z^{*}<z<1,{\frac{dF(X)}{dx}|}_{x=0}>0$ and ${\frac{dF(X)}{dx}|}_{x=1}<0.x=1$ is the equilibrium point of the evolutionary game, and the final result is to choose a strong regulatory strategy.

**Village collective response behaviour.** The expected utilities of positive and negative village collectives’ response are *U*_1*y*_,*U*_2*y*_, and the average expected utilities are $\bar{U_{y}}$. The expressions are:

$$U_{1y}=xz(R_{v}-C_{v}-\Delta V+B_{v1}-M_{p1}+B_{v2})+x(1-z)(R_{v}-C_{v}-\Delta V+B_{v1})+(1-x)z(R_{v}-C_{v}-\Delta V+B_{v1}-M_{p1}+B_{v2})+(1-x)(1-z)(R_{v}-C_{v}-\Delta V+B_{v1})$$ (5)

$$U_{2y}=xz(R_{v}-C_{v}-D_{v1}-\alpha T_{p2}-F_{v}-\alpha D_{v2})+x(1-z)(R_{v}-C_{v}-D_{v1}-F_{v})+(1-x)z(R_{v}-C_{v}-D_{v1}-\alpha T_{p2}-\alpha F_{v}-\alpha D_{v2})+(1-x)(1-z)(R_{v}-C_{v}-D_{v1})$$ (6)

$$\bar{U_{y}}=yU_{1y}+(1-y)U_{2y}$$ (7)

The replicated dynamic equation *F*(*Y*) for the village collectives choosing a positive response strategy is as follows:

$$F(Y)=\frac{dy}{dt}=y(U_{1y}-\bar{U_{y}})=y(1-y)[\left(B_{v1}+D_{v1}-\Delta V)-xz\alpha F_{v}+xF_{v}+z(-M_{p1}+\alpha T_{p2}+\alpha F_{v}+B_{v2}+\alpha D_{v2}\right)]$$ (8)

Let *F*(*Y*) = 0 and note $z^{*}=\frac{(\Delta V-B_{v1}-D_{v1})-xF_{v}}{(-M_{p1}+\alpha T_{p2}+\alpha F_{v}+B_{v2}+\alpha D_{v2})-x\alpha F_{v}}$, the evolutionary stability point of village collectives response is analysed as follows:

(1) If *z* = z*, then the replicated dynamic equation *F*(*Y*)≡0 and all response decisions *y* are evolutionary equilibrium points. Regardless of the initial proportion of village collectives choosing ‘positive response’ or ‘negative response’, the outcome does not change over time.
(2) If *z*≠z*. Let *F*(*Y*) = 0, then *y* = 0 and *y* = 1 are two possible equilibrium points. From the stability condition of the replicated dynamic equation, it can be deduced that if $\frac{dF(Y)}{dy}=(1-2y)[\left(B_{v1}+D_{v1}-\Delta V)-xz\alpha F_{v}+xF_{v}+z(-M_{p1}+\alpha T_{p2}+\alpha F_{v}+B_{v2}+\alpha D_{v2}\right)]<0$, the result is the stable point of the evolutionary game. In particular, two different cases were discussed:
1) When $0<z<z^{*},\;{\frac{dF(Y)}{dy}|}_{y=0}<0$ and ${\frac{dF(Y)}{dy}|}_{y=1}>0.y=0$ is the equilibrium point of the evolutionary game, and the final result is to choose the negative response strategy.
2) When $z^{*}<z<1,\;{\frac{dF(Y)}{dy}|}_{y=0}>0$ and ${\frac{dF(Y)}{dy}|}_{y=1}<0$. *y* = 1 is the equilibrium point of the evolutionary game, and the final result is to choose the positive response strategy.

**Farmer participation behaviour.** The expected utilities of active and passive farmers participation are *U*_1*z*_,*U*_2*z*_ and the average expected utilities are $\bar{U_{z}}$. The expressions are:

$$U_{1z}=\&xy(U_{1}-C_{p1}+M_{p1})+x(1-y)(U_{2}-C_{p1}-C_{p2}+{\alpha T}_{p1}+\alpha T_{p2})+(1-x)y(U_{1}-C_{p1}+M_{p1})+(1-x)(1-y)(U_{2}-C_{p1}-C_{p2}+{\alpha T}_{p1}+\alpha T_{p2})$$ (9)

$$U_{2z}=xy(U_{1}-\beta F_{p})+x(1-y)(U_{2}-\beta F_{p})+(1-x)y(U_{1})+(1-x)(1-y)(U_{2})$$ (10)

$$\bar{U_{z}}=zU_{1z}+(1-z)U_{2z}$$ (11)

The replicated dynamic equation *F*(*Z*) for the farmer choosing an active participation strategy is as follows:

$$F(Z)=\frac{dz}{dt}=z(U_{1z}-\bar{U_{z}})=z(1-z)[\left({\alpha T}_{p1}+\alpha T_{p2}-C_{p1}-C_{p2})+x\beta F_{p}+y(M_{p1}+C_{p2}-{\alpha T}_{p1}-\alpha T_{p2}\right)]$$ (12)

Let *F*(*Z*) = 0 and note $x^{*}=\frac{\left(C_{p1}+C_{p2}-{\alpha T}_{p1}-\alpha T_{p2})-y(M_{p1}+\beta F_{p}+C_{p2}-{\alpha T}_{p1}-\alpha T_{p2}\right)}{\beta F_{p}}$, the evolutionary stability point of farmer participation is analysed as follows:

(1) If *x* = x*, then the replicated dynamic equation *F*(*Z*) ≡ 0 and all participation decisions *z* are evolutionary equilibrium points. Regardless of the initial proportion of farmers choosing ‘active participation’ or ‘passive participation’, the outcome does not change over time.
(2) If *x*≠x*. Let *F*(*Z*) = 0, then z = 0 and z = 1 are two possible equilibrium points. From the stability condition of the replicated dynamic equation, it can be deduced that if $\frac{dF(Z)}{dz}=(1-2z)[\left({\alpha T}_{p1}+\alpha T_{p2}-C_{p1}-C_{p2})+x\beta F_{p}+y(M_{p1}+C_{p2}-{\alpha T}_{p1}-\alpha T_{p2}\right)]<0$, the result is the stable point of the evolutionary game. In particular, two different cases were discussed:
1) When $0<x<x^{*},\;{\frac{dF(Z)}{dz}|}_{z=0}<0$ and ${\frac{dF(Z)}{dz}|}_{z=1}>0$. *z* = 0 is the equilibrium point of the evolutionary game, and the final result is to choose the passive participation strategy.
2) When $x^{*}<x<1,\;{\frac{dF(Z)}{dz}|}_{z=0}>0$ and ${\frac{dF(Z)}{dz}|}_{z=1}<0$. *z* = 1 is the equilibrium point of the evolutionary game, and the final result is to choose the active participation strategy.

### Multiple subject

If we construct the dynamic equation of the three-party game replication as shown in Eq (13), the equilibrium points of the system can be obtained as $E_{1}(0,0,0),\;E_{2}(1,0,0),\;E_{3}(0,1,0),\;E_{4}(0,0,1),\;E_{5}(1,1,0),\;E_{6}(1,0,1),\;E_{7}(0,1,1)$ and *E*_8_(1,1,1), respectively.


$$\left\{ \begin{array}{l} F(X)=x(1-x)[\left(T_{g2}-T_{g1}-\Delta G+F_{v}+A+\beta F_{p})+yz\alpha F_{v}-yF_{v}+z(B_{g}+\alpha D_{g}-\alpha F_{v}-\beta F_{p}\right)]\\ F(Y)=y(1-y)[\left(B_{v1}+D_{v1}-\Delta V)-xz\alpha F_{v}+xF_{v}+z(-M_{p1}+\alpha T_{p2}+\alpha F_{v}+B_{v2}+\alpha D_{v2}\right)]\\ F(Z)=z(1-z)[\left({\alpha T}_{p1}+\alpha T_{p2}-C_{p1}-C_{p2})+x\beta F_{p}+y(M_{p1}+C_{p2}-{\alpha T}_{p1}-\alpha T_{p2}\right)] \end{array}\right.$$ (13)

Furthermore, the equilibrium points are introduced separately into the Jacobian matrix, and by Lyapunov’s stability theorem, it is ESS if the characteristic roots are all negative real roots. The Jacobian matrix—shown in Eq (14)—is unstable if any of the characteristic roots of the Jacobian matrix is greater than 0.


$$J=\left(\begin{array}{ccc} \frac{\partial F(X)}{\partial x} & \frac{\partial F(X)}{\partial y} & \frac{\partial F(X)}{\partial z}\\ \frac{\partial F(Y)}{\partial x} & \frac{\partial F(Y)}{\partial y} & \frac{\partial F(Y)}{\partial z}\\ \frac{\partial F(Z)}{\partial x} & \frac{\partial F(Z)}{\partial y} & \frac{\partial F(Z)}{\partial z} \end{array}\right)=\left[\begin{array}{ccc} a_{11} & a_{12} & a_{13}\\ a_{21} & a_{22} & a_{23}\\ a_{31} & a_{32} & a_{33} \end{array}\right]$$ (14)

$$a_{11}=(1-2x)[\left(T_{g2}-T_{g1}-\Delta G+F_{v}+A+\beta F_{p})+yz\alpha F_{v}-yF_{v}+z(B_{g}+\alpha D_{g}-\alpha F_{v}-\beta F_{p}\right)]$$

$$a_{12}=x(1-x)[z\alpha F_{v}-F_{v}]$$

$$a_{13}=x(1-x)[y\alpha F_{v}+(B_{g}+\alpha D_{g}-\alpha F_{v}-\beta F_{p})]$$

$$a_{21}=y(1-y)[-z\alpha F_{v}+F_{v}]$$

$$a_{22}=(1-2y)[\left(B_{v1}+D_{v1}-\Delta V)-xz\alpha F_{v}+xF_{v}+z(-M_{p1}+\alpha T_{p2}+\alpha F_{v}+B_{v2}+\alpha D_{v2}\right)]$$

$$a_{23}=y(1-y)[-x\alpha F_{v}-M_{p1}+\alpha T_{p2}+\alpha F_{v}+B_{v2}+\alpha D_{v2}]$$

$$a_{31}=z(1-z)\beta F_{p}$$

$$a_{32}=z(1-z)[M_{p1}+\beta F_{p}+C_{p2}-{\alpha T}_{p1}-\alpha T_{p2}]$$

$$a_{33}=(1-2z)[\left({\alpha T}_{p1}+\alpha T_{p2}-C_{p1}-C_{p2})+x\beta F_{p}+y(M_{p1}+C_{p2}-{\alpha T}_{p1}-\alpha T_{p2}\right)]$$

The content and subjects of this paper are derived from the current development in China.

The following conditions must be met to make the hypotheses and matrix modelling realistic. (1) The central government subsidy is greater than the incremental cost of local governments adopting strong regulation, i.e. *A*>Δ*G*. (2) Regardless of the strategy adopted by local governments, the difference in subsidy given to rural areas will not be higher than the amount of the penalty, i.e. *F*_*v*_>*T*_*g*1_−*T*_*g*2_. (3) The incremental cost minus the social benefit when village collectives respond positively is greater than the social loss when they respond negatively, indicating the need to rely on the government to promote ANSPC, i.e. Δ*V*−*B*_*v*1_>*D*_*v*1_. (4) The cost of farmers’ active participation is greater than the compensation received, i.e. *C*_*p*1_+*C*_*p*2_>*T*_*p*1_+*T*_*p*2_; therefore, the stability conditions of the eight equilibrium points are analysed, as shown in Table 3.

**Table 3:** Characteristic roots and stability conditions of the equilibrium points.

Equilibrium points	Characteristic roots (*λ*_1_,*λ*_2_,*λ*_3_)	Symbol	Stability conditions
*E*_1_(0,0,0)	*T*_*g*2_−T_*g*1_−Δ*G*+*A*+*F*_*v*_+*βF*_*p*_, *B*_*v*1_+*D*_*v*1_−Δ*V*, *αT*_*p*1_+*αT*_*p*2_−*C*_*p*1_−*C*_*p*2_	+ - -	Instability point
*E*_2_(1,0,0)	*T*_*g*1_−T_*g*2_−*A*+Δ*G*−*F*_*v*_−*βF*_*p*_, *B*_*v*1_+*D*_*v*1_−Δ*V*+*F*_*v*_, *αT*_*p*1_+*αT*_*p*2_−*C*_*p*1_−*C*_*p*2_+*βF*_*p*_	- * *	*D*_*v*1_+*F*_*v*_<Δ*V*−*B*_*v*1_, *αT*_*p*1_+*αT*_*p*2_+*βF*_*p*_*<C*_*p*1_+*C*_*p*2_
*E*_3_(0,1,0)	*T*_*g*2_−T_*g*1_−Δ*G*+*A*+*βF*_*p*_, Δ*V*−*B*_*v*1_−*D*_*v*1_, *M*_*p*1_−*C*_*p*1_	* + *	Instability point
*E*_4_(0,0,1)	*T*_*g*2_−T_*g*1_−Δ*G*+*A*+*F*_*v*_+*B*_*g*_+*αD*_*g*_−*αF*_*v*_, *B*_*v*1_+*D*_*v*1_−Δ*V*−*M*_*p*1_+*αT*_*p*2_+*αF*_*v*_+*B*_*v*2_+*αD*_*v*2_, *C*_*p*1_+*C*_*p*2_−*αT*_*p*1_−*αT*_*p*2_	* * +	Instability point
*E*_5_(1,1,0)	*T*_*g*1_−T_*g*2_+Δ*G*−A−*βF*_*p*_, Δ*V*−*B*_*v*1_−*D*_*v*1_−*F*_*v*_, *M*_*p*1_+*βF*_*p*_−*C*_*p*1_	* * *	*T*_*g*1_−T_*g*2_<*A*−Δ*G*+*βF*_*p*_, Δ*V*−*B*_*v*1_<*D*_*v*1_+*F*_*v*_, *βF*_*p*_+*M*_*p*1_<*C*_*p*1_
*E*_6_(1,0,1)	*T*_*g*1_−T_*g*2_+Δ*G*−A+*αF*_*v*_−*F*_*v*_−*B*_*g*_−*αD*_*g*_, $B_{v1}+D_{v1}-\Delta V-M_{p1}+\alpha T_{p2}+F_{v}+B_{v2}+\alpha D_{v2}$, $C_{p1}+C_{p2}-{\alpha T}_{p1}-\alpha T_{p2}-\beta F_{p}$	* * *	$T_{g1}-T_{g2}-\alpha D_{g}<A-\Delta G+F_{v}-\alpha F_{v}+B_{g}$, $D_{v1}+\alpha T_{p2}+F_{v}<\Delta V+M_{p1}-B_{v1}-B_{v2}-\alpha D_{v2}$, $C_{p1}+C_{p2}-\beta F_{p}<{\alpha T}_{p1}+\alpha T_{p2}$
*E*_7_(0,1,1)	$T_{g2}-T_{g1}-\Delta G+A+B_{g}+\alpha D_{g}$, ${\Delta V-B}_{v1}-D_{v1}+M_{p1}-\alpha T_{p2}-\alpha F_{v}-B_{v2}-\alpha D_{v2}$, *C*_*p*1_−*M*_*p*1_	* * *	$A+B_{g}<T_{g1}-T_{g2}+\Delta G-\alpha D_{g}$, $\Delta V-D_{v1}-\alpha T_{p2}-\alpha F_{v}-\alpha D_{v2}<B_{v1}+B_{v2}-M_{p1}$, *C*_*p*1_<*M*_*p*1_
*E*_8_(1,1,1)	$T_{g1}-T_{g2}+\Delta G-A-B_{g}-\alpha D_{g}$, $\Delta V-B_{v1}-D_{v1}+M_{p1}-\alpha T_{p2}-F_{v}-B_{v2}-\alpha D_{v2}$, *C*_*p*1_−*M*_*p*1_−*βF*_*p*_	* * *	$T_{g1}-T_{g2}+\Delta G-\alpha D_{g}<A+B_{g}$, $\Delta V-D_{v1}-\alpha T_{p2}-F_{v}-\alpha D_{v2}<B_{v1}+B_{v2}-M_{p1}$, *C*_*p*1_−*βF*_*p*_<*M*_*p*1_

Table 3 indicates five possible ESS points. In this paper, the ESS points are classified and discussed according to the ANSPC stage requirements.

**Stage of awareness raising. Corollary 1.** Under the conditions *D*_*v*1_+*F*_*v*_<Δ*V*−*B*_*v*1_and ${\alpha T}_{p1}+\alpha T_{p2}<C_{p1}+C_{p2}-\beta F_{p},\;E_{2}(1,0,0)$ is the evolutionary stable point.

**Proof of corollary 1.** Table 3 shows that the equation satisfies the stability condition for the equilibrium point *E*_2_(1,0,0).

**Fig 1:**
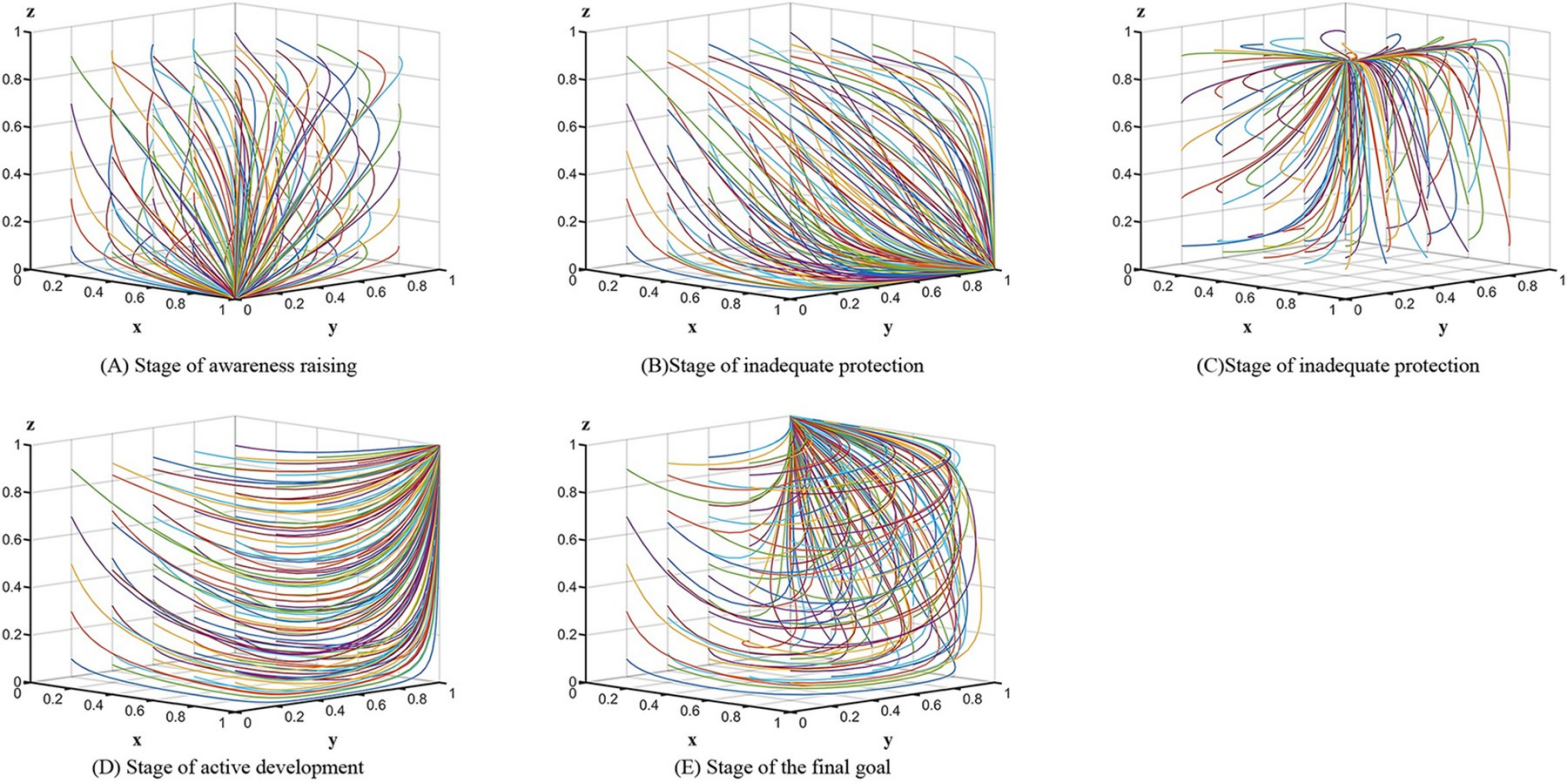
Evolutionary paths in different stages.

Corollary 1 suggests that local governments value ANSPC at this stage. When local governments choose a strong regulatory strategy, they can receive subsidies from the central government to compensate for the extra costs paid. Simultaneously, local governments fine the subjects of negative behaviour as part of their fiscal revenue. Therefore, the local government prefers the strong regulation strategy, where *x* = 1. The village collectives have two choices; the first is to respond negatively, generating social losses with fines from the local government. The second is to respond positively, generating the difference between the incremental costs of pollution control and the social benefits. Since the latter accrues greater loss than pollution control, the village collectives choose to respond negatively, where *y* = 0. For the farmers, the fine for polluting is smaller and the difference between the benefits and costs of active participation is large; thus, they choose to participate passively, where *z* = 0. This stage is the primary level of ANSPC, which is a poor condition. The evolutionary path of the three-party game is shown in Fig 1(A).

**Stage of inadequate protection. Corollary 2.** Under the conditions $T_{g1}-T_{g2}<A-\Delta G+\beta F_{p},\;\Delta V-D_{v1}-F_{v}<B_{v1}$ and *F*_*p*_+*M*_*p*1_<*C*_*p*1_,*E*_5_(1,1,0) is the evolutionary stable point.

**Proof of corollary 2.** Table 3 shows that the equation satisfies the stability condition for the equilibrium point *E*_5_(1,1,0).

Corollary 2 suggests that at this stage, the difference between the social benefit and the village collective’s incremental cost is less than the sum of the fine and social loss, so a positive response is chosen, where *y* = 1. The net benefit to the local government is the central subsidy plus the fine minus the subsidy paid, which is greater than the incremental cost of strong regulation, so a strong regulation strategy is chosen, where *x* = 0. The fine farmers’ passive participation is less than the net cost of active participation (cost minus reward), so passive participation is chosen, where *z* = 0. The evolution path is shown in Fig 1(B).

**Corollary 3.** Under the conditions $T_{g1}-T_{g2}-\alpha D_{g}<A-\Delta G+F_{v}-\alpha F_{v}+B_{g},\;D_{v1}+\alpha T_{p2}+F_{v}<\Delta V+M_{p1}-B_{v1}-B_{v2}-\alpha D_{v2}$ and $C_{p1}+C_{p2}-\beta F_{p}<{\alpha T}_{p1}+\alpha T_{p2},\;E_{6}(1,0,1)$ is the evolutionary stable point.

**Proof of corollary 3.** Table 3 shows that the equation satisfies the stability condition for the equilibrium point *E*_6_(1,0,1).

Corollary 3 suggests that the farmers’ gain comes from the compensation for reporting to the village collective and the local government’s reward for reporting, which is greater than the cost paid minus the fine, so the farmers choose active participation, where *z* = 1. The extra gain from strong regulation by the local government is greater than the loss from weak regulation, so the strong regulation strategy is chosen, where *x* = 1. Similarly, farmers’ participation affects village collectives; however, the net cost to village collectives is greater if they respond positively, so they choose to respond negatively, where *y* = 0. The evolution path is shown in Fig 1(C).

**Stage of active development. Corollary 4.** Under the conditions $T_{g1}-T_{g2}+\Delta G-\alpha D_{g}<A+B_{g},\;V-D_{v1}-\alpha T_{p2}-F_{v}-\alpha D_{v2}<B_{v1}+B_{v2}-M_{p1}$ and *C*_*p*1_—*βF*_*p*_<*M*_*p*1_,*E*_8_(1,1,1) is the evolutionary stable point.

**Proof of corollary 4.** Table 3 shows that the equation satisfies the stability condition for the equilibrium point *E*_8_(1,1,1).

Corollary 4 suggests that at this stage, the local government receives central subsidies with additional benefits greater than the incremental costs and therefore chooses a robust regulatory strategy, where *x* = 1. The sum of the benefits from the village collectives minus the subsidies to the farmers is greater than the incremental costs, and the positive response strategy is chosen, where *y* = 1. The benefits of the farmers’ active participation are greater than the costs; therefore, the active participation strategy is chosen, where *z* = 1. This stage is an advanced level of ANSPC, and all three subjects obtained benefits. The evolution path is shown in Fig 1(D).

**Stage of the final goal. Corollary 5.** Under the conditions $A+B_{g}<T_{g1}-T_{g2}+\Delta G-\alpha D_{g},\;\Delta V-D_{v1}-\alpha T_{p2}-\alpha F_{v}-\alpha D_{v2}<B_{v1}+B_{v2}-M_{p1}$ and *C*_*p*1_<*M*_*p*1_,*E*_7_(0,1,1) is the evolutionary stable point.

**Proof of corollary 5.** Table 3 shows that the equation satisfies the stability condition for the equilibrium point *E*_7_(0,1,1).

Corollary 5 suggests that at this stage, the sum of subsidies and benefits received by the local government is less than the incremental costs, and a weak regulation strategy is chosen, where *x* = 0. The sum of the social and reputational benefits of village collectives is greater and can cover the costs, and a positive response is chosen, where *y* = 1. The reward for farmers’ active participation is greater than the cost of participation; thus, an active participation strategy is chosen, where *z* = 1. This stage is the ultimate goal to be achieved. The evolution path is shown in Fig 1(E).

### Numerical simulation

Numerical simulations were conducted using MATLAB 2022a to observe the dynamic evolution more intuitively and further analyse the impact of changes in various influencing factors on the subject’s decision. Since ANSPC in China is currently in a situation of “strong regulation, positive response, and active participation” for a long time [45], the evolutionary stability conditions are $T_{g1}-T_{g2}+\Delta G-\alpha D_{g}<A+B_{g},\;\Delta V-D_{v1}-\alpha T_{p2}-F_{v}-\alpha D_{v2}<B_{v1}+B_{v2}-M_{p1},\;C_{p1}-\beta F_{p}<M_{p1}$.

Regarding parameter assignment, this paper selects the List of Advanced Counties in the National Village Cleanup Initiative in 2021 (hereinafter referred to as ‘the List’) published by the Ministry of Agriculture and Rural Development as the initial sample set. The List recognises 98 advanced counties with excellent performance in ANSPC in 31 provinces, autonomous regions and municipalities directly under the central government; therefore, the advanced counties in the List are representative and typical nationwide. Second, this paper uses a combination of random and stratified sampling, from which 20 advanced counties were selected for data collection and survey. Again, this paper compiles the actual values of documents such as official documents, reports and information on ANSPC parameters of the 20 advanced counties through the Internet and yearbooks. These include, but are not limited to, model farmer profiles, public government reports on financial support, ANSPC costs, fines and revenues. To ensure the accuracy of the data, this paper also uses telephone interviews with demonstration village governments, leaders of village collectives, and experts in related fields. Finally, this paper summarises the actual values of different demonstration farmers and takes the average, scales the parameters equally and derives the numerical assumptions, as shown in Table 4.

**Table 4:** Parameter assignment.

Δ*G*	*T*_*g*1_	*T*_*g*2_	*F*_*v*_	*A*	*T*_*p*1_	*F*_*p*_	*B*_*g*_	*D*_*g*_	Δ*V*	*B*_*v*1_	*D*_*v*1_	*T*_*p*2_	*B*_*v*2_	*D*_*v*2_	*α*	*β*	*M*_*p*1_	*C*_*p*1_	*C*_*p*2_
5	6	5	3	6	3	2	1	2	5	3	1	3	1	2	0.5	0.5	2	2.5	5

### Effect of initial willingness on the evolutionary results

**Fig 2:**
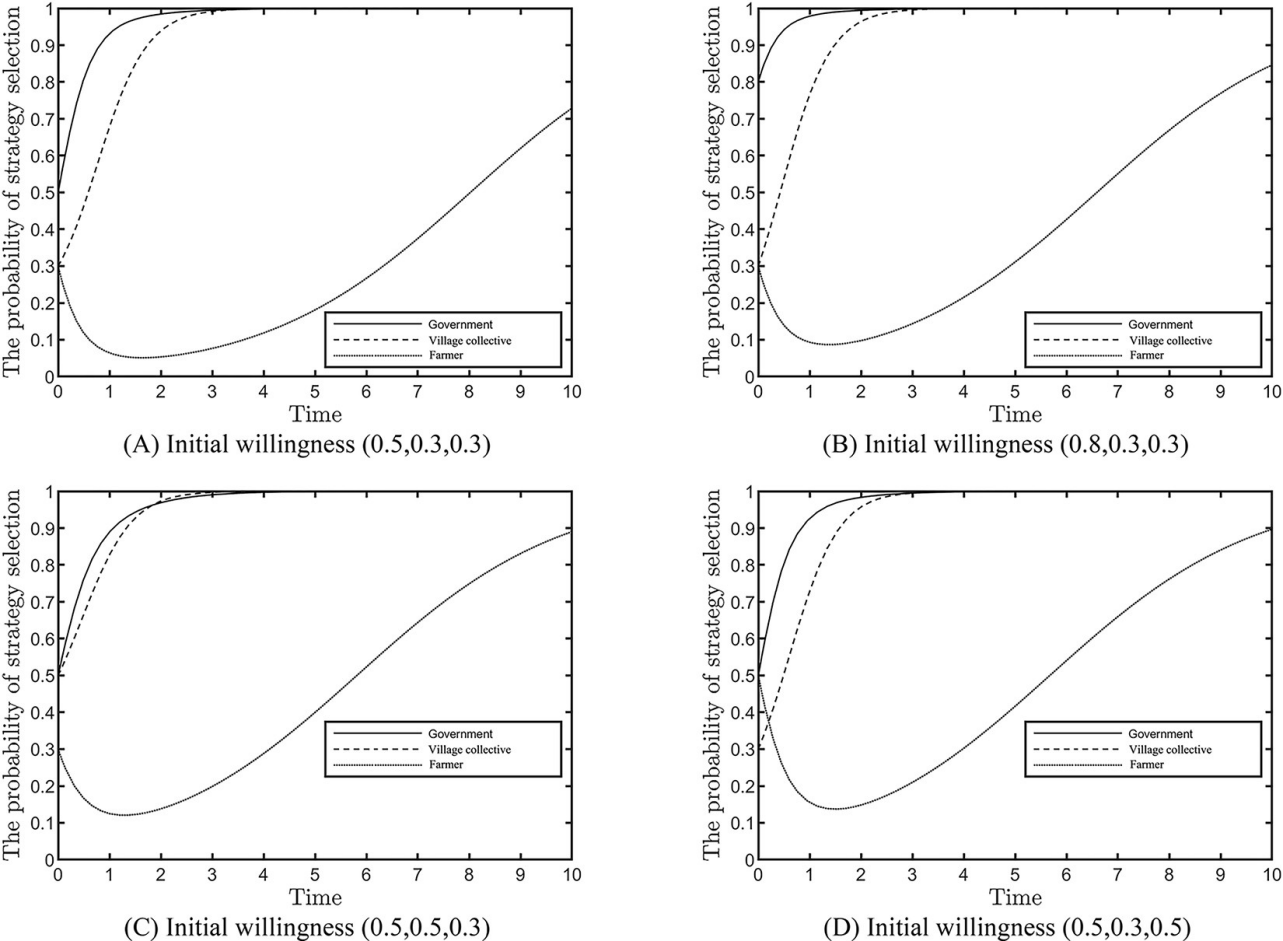
Effect of initial willingness on evolution results.

The influence of the subject’s initial readiness on the evolution outcomes—with all other parameters held constant—is shown in Fig 2. It is assumed that the initial willingness of the local government to regulate strongly is 0.5, and the initial willingness of the village collectives to actively respond and the farmers to actively participate is 0.3. The game results of the three parties are ‘strong regulation, positive response and active participation’; however, differences exist in the speed of convergence, with local government converging the fastest, followed by village collectives and farmers converging the slowest, as shown in Fig 2(A). Fig 2(B) to 2(D) increase the initial willingness of the three subjects, respectively, and the results indicate the following. (1) The level of initial willingness does not affect the convergence results and has the characteristics of path dependence, but the higher the initial willingness, the shorter the convergence time to stability. (2) When the initial willingness of village collectives to respond positively increases, it can shorten its evolutionary stabilisation time. Meanwhile, the speed of convergence of strong local government regulation is less reduced but can significantly reduce the evolutionary time of farmers. (3) The initial willingness of farmers to actively participate is increased, which has less impact on the speed of convergence of other issues.

### Effect of central subsidy on the evolutionary results

**Fig 3:**
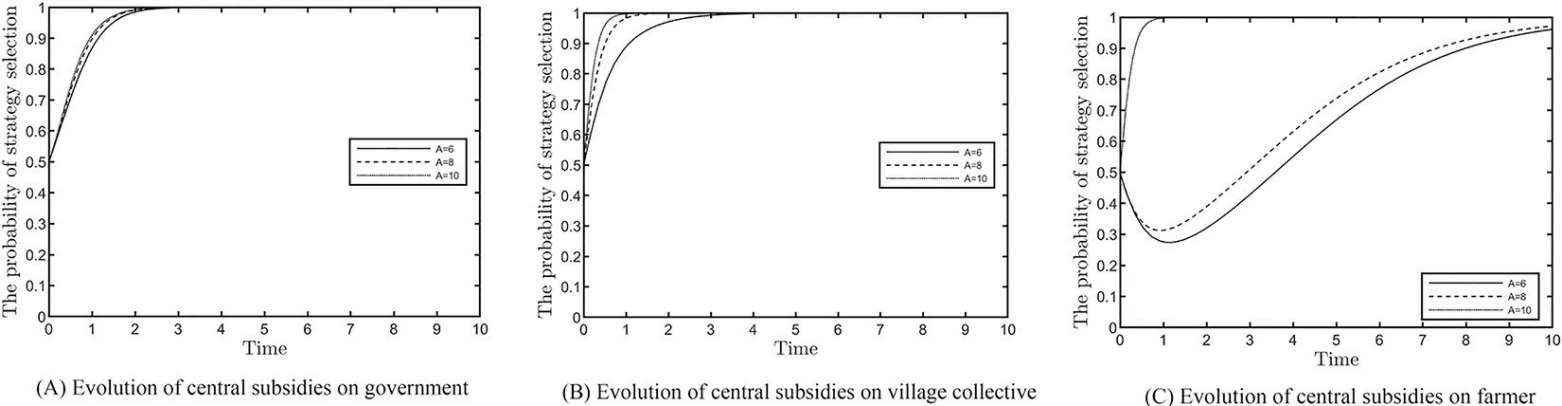
Effect of central subsidies on evolution results.

This section analyses the effect of the central subsidy on the dynamic evolution results of the three subjects, as shown in Fig 3. Based on satisfying the stability point constraint, values of 6, 8 and 10 are assigned to *A*. The increase in the central subsidy significantly increases the convergence rate for local governments that choose a robust regulatory strategy, as shown in Fig 3(A), indicating that the central subsidy is one of the incentives for local governments to adopt regulatory policies. Local governments punish village collectives and farmers who adopt negative strategies; both accelerate the rate of development to reduce costs, but the development is less efficient. Fig 3(B) also shows that the central subsidy has little effect on the stability outcome of village collectives. Fig 3(C) shows that there is a threshold for the effect of the central subsidy on farmers, and when the amount of subsidy exceeds the threshold, it significantly increases farmers’ evolution rate to the stabilisation point.

### Effect of fine on the evolutionary results

**Fig 4:**
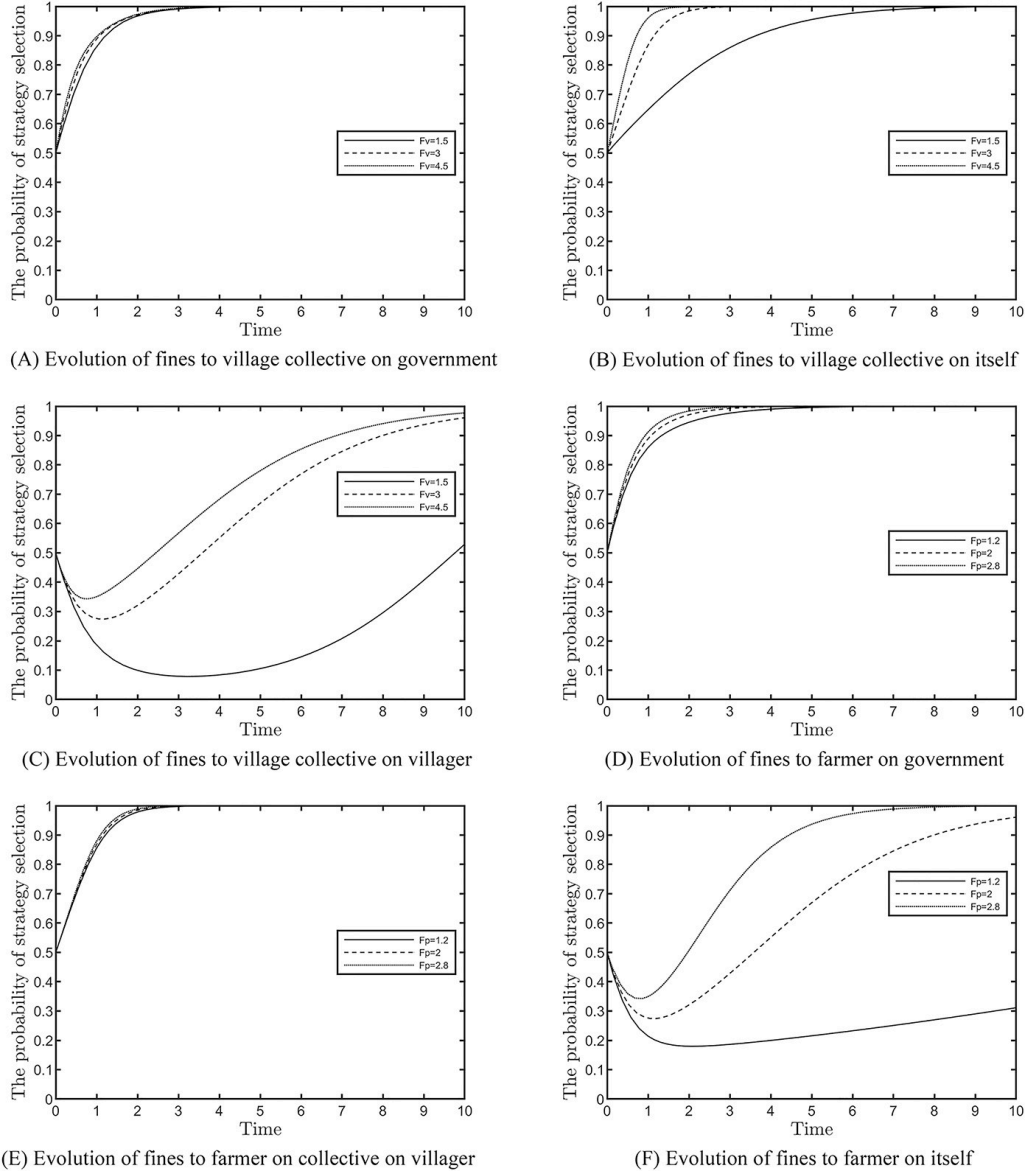
Effect of fines on evolution results.

This section analyses the impact of fines’ intensity on the tripartite subjects’ evolutionary outcomes, as shown in Fig 4. Among them, Fig 4(A)–4(C) show the fines to village collectives, and Fig 4(D)–4(F) show the fines to farmers. Based on the fulfilment of the constraints, *F*_*v*_ is assigned 1.5, 3 and 4.5 and *F*_*p*_ is assigned 1.2, 2 and 2.8, respectively. Although the government increases the rate of development of village collectives and farmers by increasing fines, both have a weak economic base and are highly dependent on ANSPC subsidies; therefore, the change in the slope of the curves in Fig 4(A) and 4(D) show that fines have a negligible impact on the rate of development stabilisation. Fig 4(B) shows that when the fine is increased from 1.5 to 4.5, the increasing cost reverses the positive response of village collectives and encourages the active participation of farmers, as shown in Fig 4(C). Although the increasing intensity of fines accelerates the farmers’ evolution, the village collectives’ sense of loss is weaker at this time, as shown in Fig 4(E) and 4(F).

### Effect of social benefits of village collectives on the evolutionary results

**Fig 5:**
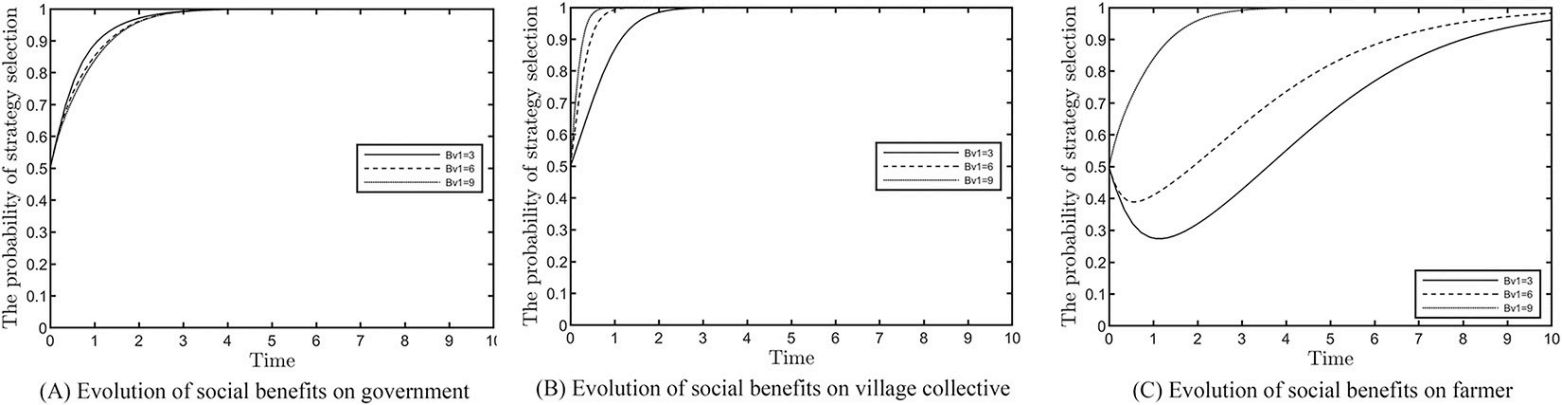
Effect of social benefits on evolution results.

This section analyses the impact of village collectives’ social effectiveness on the evolutionary outcomes, as shown in Fig 5. Based on satisfying the constraints, values of 3, 6 and 9 are assigned to *B*_*v*1_. Fig 5(A) shows that when the social benefits of village collectives increase, the administrative coercion of local government is alleviated, and the period of strong regulatory stability is extended to period 3. Fig 5(B) shows that as the social benefits from the positive response of village collectives increase, the evolutionary stability results are improved from period 2 to period 1, and the evolutionary speed increases. Fig 5(C) indicates that the farmers tend to actively participate more quickly due to the increase in all benefits.

### Effect of farmers reporting on the evolutionary results

**Fig 6:**
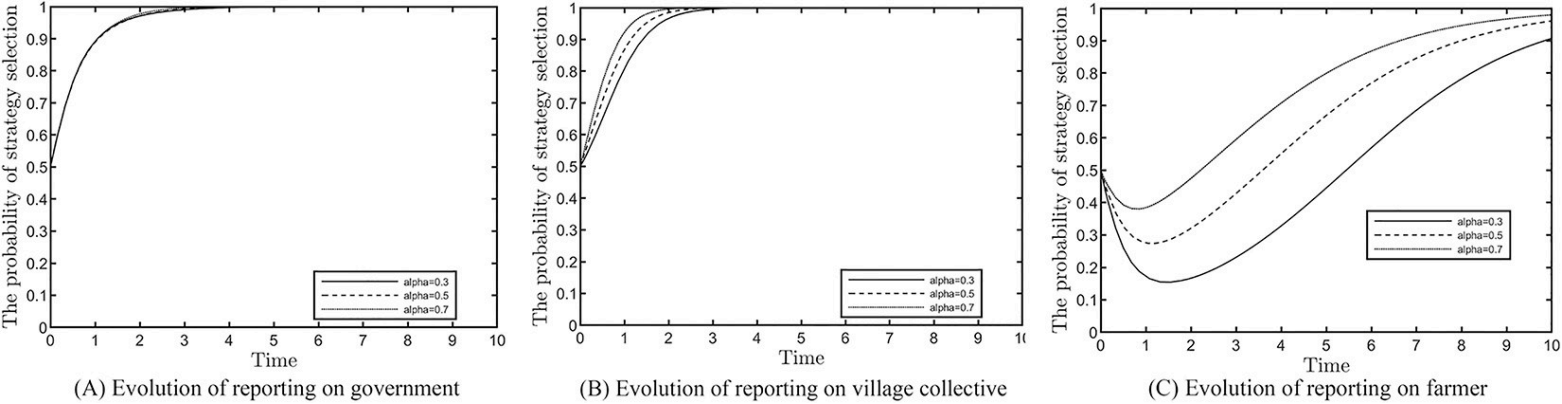
Effect of reporting on evolution results.

This section analyses the impact of the probability of farmers reporting and succeeding on the evolutionary results, as shown in Fig 6. Based on the fulfilment of the constraints, values of 0.3, 0.5 and 0.7 are assigned to *α*. In Fig 6(A), the three curves generally overlap regardless of the change in probability, indicating that the change in the probability of farmers reporting and being successful has little effect on the local government’s decision. Fig 6(B) indicates that village collectives are supervised by both the local government and the farmers, which increases the cost of negative response and implements positive response strategies. Fig 6(C) shows that as the probability of farmers reporting and being successful increases, the trend of their active participation stabilises.

### Effect of reputation change on evolutionary results

**Fig 7:**
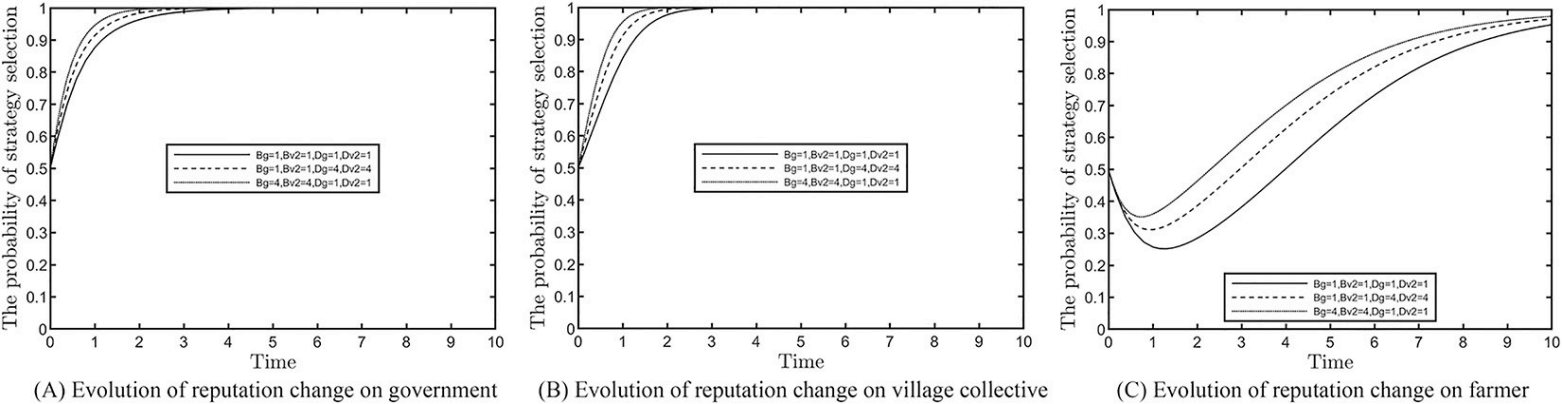
Effect of reputation change on evolution results.

This section analyses the impact of the reputation change on the evolutionary results of the tripartite subjects under the active participation of the farmers, as shown in Fig 7. Based on the constraints, the combinations $B_{g}=B_{v2}=D_{g}=D_{v2}=1:\;B_{g}=B_{v2}=1,\;D_{g}=D_{v2}=4;\;B_{g}=B_{v2}=4,\;D_{g}=D_{v2}=1$ are constructed. We define ‘reputation change’ to include the reputation gain of local governments and village collectives in the ‘strong regulation, positive response’ state and the reputation loss in the ‘weak regulation, negative response’ state. The results in Fig 7(A)–7(C) indicate that farmers’ active participation can accelerate the evolutionary efficiency of local governments and village collectives, both in terms of reputation gain and reputation loss. Simultaneously, the evolutionary efficiency is higher when the additional gains are greater than the losses.

### Conclusion and policy recommendations

Continuous promotion of ANSPC is inevitable for achieving high-quality development and protection of farmland systems. However, the internal logic of stakeholders’ involvement in ANSPC is still unclear, leading to opportunistic behaviour. Therefore, it is necessary to explore the learning behaviours and strategic interactions of different subjects to provide decision-making reference for the government formulating ANSPC programs. This paper constructs an evolutionary game model involving three subjects—local government, village collectives and farmers—and explores the conditions of equilibrium and stability points of the system. The main conclusions are as follows.

(1) Local governments play a leading role in ANSPC, but village collectives are the key to the system. First, the higher the initial willingness of local governments to strong regulation, the faster village collectives and farmers reach stability point. In different cases, local governments reach stability at an extremely speed, village collectives follow slightly behind, whereas evolutions of farmers are the slowest, indicating that policies may not directly increase farmers ’ willingness to participate. The interdependence mechanism of the three subjects can be summarized in two cycles. As a supplement, village collectives connect with farmers more directly and can convey government information to farmers, ultimately significantly increasing their willingness to participate. Meanwhile, village collectives can reduce the willingness of the local government to strong regulation, which in turn promotes the evolution of ANSPC to the next stage. In summary, although any subject’s willingness change will affect the speed of system evolution to stability, village collectives link local governments and farmers, which enhances the enforceability of ANSPC.

(2) Five evolutionary stability points exist in ANSPC, reflecting the results of the subject’s strategic choices. According to the subject interaction, it can be divided into four stages: awareness raising, inadequate protection, active development and final goal. Specifically, *E*_2_(1,0,0) is the stability point of the first stage, where the local government regulates ANSPC under central subsidy, but other subjects have a wait-and-see attitude. As the policy gradually supplements, *E*_5_(1,1,0) and *E*_6_(1,0,1) are the second stage stability points. Subjects choose appropriate strategies based on the comparison of costs and benefits, yet cannot satisfy all subjects at the same time. *E*_8_(1,1,1) and *E*_7_(0,1,1) represent the ideal stability point of the stage of active development and the stage of final goal, respectively. ANSPC will be efficient if the net benefits to village collectives and farmers are guaranteed. More importantly, the local government ultimately chooses to relax or even withdraw from the regulation because the spontaneous governance behaviour of the two subjects reduces the cost of supervision and implementation.

(3) Some parameters can have significantly different impacts on subjects’ strategy choices, including the central subsidy, the size of the fine, the social benefit, the probability of reporting and success and the reputation change. Central subsidies are one of the driving forces for local governments to adopt a robust regulatory strategy, while they have less impact on the evolutionary efficiency of village collectives and farmers. Village collectives and farmers are most sensitive to changes in social efficiency. The decision-making time of village collectives is significantly shortened. Moreover, when the social benefits exceed the threshold value, the farmers’ decision path changes, which is manifested in the change in the farmers’ pre-programming behaviour from resistance to acceptance. In addition, regardless of the policy choices of local governments and village collectives, the reputational change due to farmers’ active participation can lead to multiple issues in conducting ANSPC, the push effect of gains outweighs the reverse push effect of losses. However, penalties have distinctive impacts. First, local governments can improve the evolutionary efficiency of village collectives and farmers through fines; however, due to the weak economic base of rural areas, fines cannot significantly improve evolutionary efficiency of government. Second, when the fines for village collectives increase, although there is a marginal decreasing effect on evolutionary efficiency, it can achieve the goal of multi-subject co-governance. Finally, increasing the fines for farmers may increase their willingness to participate, but it cannot effectively increase the willingness of village collectives.

To encourage multi-stakeholders to realise the long-term co-management of ANSPC, this paper also proposes the following recommendations: (1) Create a good atmosphere for rural environmental governance. Disseminating relevant policies through formal and informal channels can help local governments define the scope of their functions, reduce the scepticism of village collectives and farmers and ultimately achieve a tripartite governance outcome. On the one hand, we should pay attention to the training of rural professionals and give full play to the proper leadership role of village collectives among farmers. On the other hand, village collectives should be given appropriate rights and responsibilities to reduce excessive government intervention. (2) Establish effective reward and punishment mechanisms and use policy instruments wisely. The central government should actively develop multiple funding sources. Using market-based mechanisms to establish cooperation between government and social capital to promote ANSPC is a feasible option. Since village collectives significantly influence farmers’ decisions, local governments should prioritise limited resources for village collectives, which can lead to active farmers’ participation. Village collectives should improve their monitoring system and adopt economic punishment for those who react negatively. Financial punishment should be used cautiously for farmers and it is more appropriate to establish a publicity and education system; excessive fines harm the productive lives of farmers and go against the original purpose of rural development. (3) Improve the relevant political system. Correct assessments of social benefits can help achieve a higher level of the multi-party governance system. To properly assess and enhance the potential social benefits, local governments should strengthen support for technological and human resources in rural areas. At the time of ANSPC, capital, talent, industry and other resources should be directed to rural areas. Furthermore, enterprises, research institutions and other green innovations in rural areas can properly train village collectives and farmers. (4) Establish a robust mechanism for farmers to participate and report. First, the local government should build an ANSPC information platform to reduce the cost for farmers to report. Second, it can implement a reward system for the public to report, establish a photo plus inspection programme and take the reported incidents seriously. Finally, appropriate penalties should be imposed on village collectives that respond negatively, and the effect should be compared in similar farmers to increase their initial willingness to participate.

Due to research methodology and model assumption, this study is subject to certain limitations: (1) Although the model investigates the influence of environmental and social benefits on strategic choices of ANSPC subjects, it does not measure specific values in detail. In addition, the weights of local government on economy and environment can be added to the model to analyse from a more micro perspective. (2) The research subjects only include the mutual influence of local government, village committee and villagers. In fact, subjects such as enterprises and social organisations may play equally important roles and should be considered in the theoretical framework.

## Supporting information

S1 File (PDF)

## References

[1] Sardaro R, La Sala P, Roselli L. How does the land market capitalize environmental, historical and cultural components in rural areas? Evidences from Italy. J Environ Manage. 2020;269:110776. doi: 10.1016/j.jenvman.2020.110776 32425172

[2] Basu NB, Van Meter KJ, Byrnes DK, Van Cappellen P, Brouwer R, Jacobsen BH, et al. Managing nitrogen legacies to accelerate water quality improvement. Nat Geosci. 2022;15(2):97–105.

[3] Cristan R, Aust WM, Bolding MC, Barrett SM, Munsell JF, Schilling E. Effectiveness of forestry best management practices in the United States: Literature review. Forest Ecol Manag. 2016;360:133–51.

[4] Sabo RD, Clark CM, Compton JE. Considerations when using nutrient inventories to prioritize water quality improvement efforts across the US. Environmental research communications. 2021;3:1–13. doi: 10.1088/2515-7620/abf296 36457483 PMC9709726

[5] Wester J, Turffs D, McEntee K, Pankow C, Perni N, Jerome J, et al. Agriculture and downstream ecosystems in Florida: an analysis of media discourse. Environ Sci Pollut Res. 2023;30(2):3804–16. doi: 10.1007/s11356-022-22475-1 35960469

[6] Qiu HU, van Wesenbeeck CFA, van Veen WCM. Greening Chinese agriculture: can China use the EU experience? China Agric Econ Rev. 2021;13(1):63–90.

[7] Wang H, Liu C, Xiong L, Wang F. The spatial spillover effect and impact paths of agricultural industry agglomeration on agricultural non-point source pollution: A case study in Yangtze River Delta, China. J Clean Prod. 2023;401:136600.

[8] Hasan S, Hansen LB, Smart JCR, Hasler B, Termansen M. Tradeable Nitrogen Abatement Practices for Diffuse Agricultural Emissions: A ’Smart Market’ Approach. Environ Resour Econ. 2022;82(1):29–63.

[9] Wong SW, Tang BS, Liu JL, Liang M, Ho WKO. From "decentralization of governance" to "governance of decentralization": Reassessing income inequality in periurban China. Environ Plan A. 2021;53(6):1473–89.

[10] Chervier C, Amblard L, Déprés C. The conditions of emergence of cooperation to prevent the risk of diffuse pollution from agriculture: a case study comparison from France. JOURNAL OF ENVIRONMENTAL PLANNING AND MANAGEMENT. 2022;65(1):62–83.

[11] Vos J, Boelens R, Venot JP, Kuper M. Rooted water collectives: Towards an analytical framework. Ecol Econ. 2020;173.

[12] Tian G, Tsai WH. "Beautiful Countryside Construction," Policy Inspection Teams, and Grassroots Political Participation in China. J Contemp China. 2023;32(144):951–62.

[13] Amblard L, Mann C. Understanding collective action for the achievement of EU water policy objectives in agricultural landscapes: Insights from the Institutional Design Principles and Integrated Landscape Management approaches. Environ Sci Policy. 2021;125:76–86.

[14] Patterson JJ, Smith C, Bellamy J. Enabling and Enacting ’Practical Action’ in Catchments: Responding to the ’Wicked Problem’ of Nonpoint Source Pollution in Coastal Subtropical Australia. ENVIRONMENTAL MANAGEMENT. 2015;55(2):479–95. doi: 10.1007/s00267-014-0409-5 25423950

[15] Tian M, Zheng Y, Sun X, Zheng H. A research on promoting chemical fertiliser reduction for sustainable agriculture purposes: Evolutionary game analyses involving ‘government, farmers, and consumers’. Ecol Indic. 2022;144:109433.

[16] Okumah M, Chapman PJ, Martin-Ortega J, Novo P, Ferré M, Jones S, et al. Do awareness-focussed approaches to mitigating diffuse pollution work? A case study using behavioural and water quality evidence. J Environ Manage. 2021;287. 10.1016/j.jenvman.2021.112242 33711664

[17] Feng JC, Tang YQ, Xue S, Zhang K. Study on cooperative strategies of rural water environment governance PPP project between companies and farmers from the perspective of evolutionary game. Environ Dev Sustain. 2022;24(1):138–55.

[18] Yoder L, Chowdhury RR. Tracing social capital: How stakeholder group interactions shape agricultural water quality restoration in the Florida Everglades. Land use policy. 2018;77:354–61.

[19] Szolnoki A, Mobilia M, Jiang LL, Szczesny B, Rucklidge AM, Perc M. Cyclic dominance in evolutionary games: a review. JOURNAL OF THE ROYAL SOCIETY INTERFACE. 2014;11(100). doi: 10.1098/rsif.2014.0735 25232048 PMC4191105

[20] Ajmone Marsan G, Bellomo N, Gibelli L. Stochastic evolutionary differential games toward a systems theory of behavioral social dynamics. MATHEMATICAL MODELS & METHODS IN APPLIED SCIENCES. 2016;26(6):1051–93.

[21] Carpenter S, Caraco NF, Correll DL, Howarth R, Sharpley AN, Smith V. Non-Point Pollution of Surface Waters With Phosphorus and Nitrogen. Ecological Applications. 1998;8.

[22] Bhatt A, John J. Including farmers’ welfare in a government-led sector transition: The case of Sikkim’s shift to organic agriculture. J Clean Prod. 2023;411.

[23] Qu Y, Zhang Q, Zhan L, Jiang G, Si H. Understanding the nonpoint source pollution loads’ spatiotemporal dynamic response to intensive land use in rural China. J Environ Manage. 2022;315:115066. doi: 10.1016/j.jenvman.2022.115066 35487162

[24] Blackstock KL, Ingram J, Burton R, Brown KM, Slee B. Understanding and influencing behaviour change by farmers to improve water quality. Sci Total Environ. 2010;408(23):5631–8. doi: 10.1016/j.scitotenv.2009.04.029 19464728

[25] Santos E, Carvalho M, Martins S. Sustainable Water Management: Understanding the Socioeconomic and Cultural Dimensions. Sustainability. 2023;15(17).

[26] Almulhim AI, Cobbinah PB. Urbanization-environment conundrum: an invitation to sustainable development in Saudi Arabian cities. INT J SUST DEV WORLD. 2023;30(4):359–73.

[27] Keiser DA, Kling CL, Shapiro JS. The low but uncertain measured benefits of US water quality policy. PROCEEDINGS OF THE NATIONAL ACADEMY OF SCIENCES OF THE UNITED STATES OF AMERICA. 2019;116(12):5262–9. doi: 10.1073/pnas.1802870115 30297391 PMC6431143

[28] Lai YC, Yang H, Qiu F, Dang ZX, Luo YH. Can Rural Industrial Integration Alleviate Agricultural Non-Point Source Pollution? Evidence from Rural China. Agr Basel. 2023;13(7).

[29] Kumwimba MN, Meng FG, Iseyemi O, Moore MT, Bo Z, Tao W, et al. Removal of non-point source pollutants from domestic sewage and agricultural runoff by vegetated drainage ditches (VDDs): Design, mechanism, management strategies, and future directions. Sci Total Environ. 2018;639:742–59. doi: 10.1016/j.scitotenv.2018.05.184 29803045

[30] Yi X, Lin D, Li J, Zeng J, Wang D, Yang F. Ecological treatment technology for agricultural non-point source pollution in remote rural areas of China. Environ Sci Pollut Res. 2021;28(30):40075–87. doi: 10.1007/s11356-020-08587-6 32337672

[31] Okumah M, Chapman P, Martin-Ortega J, Novo P. Mitigating Agricultural Diffuse Pollution: Uncovering the Evidence Base of the Awareness–Behaviour–Water Quality Pathway. Water. 2018;11:29.

[32] Kaczan D, Pfaff A, Rodriguez L, Shapiro-Garza E. Increasing the impact of collective incentives in payments for ecosystem services. Journal of Environmental Economics and Management. 2017;86:48–67.

[33] Huber R, Späti K, Finger R. A behavioural agent-based modelling approach for the ex-ante assessment of policies supporting precision agriculture. Ecol Econ. 2023;212:107936.

[34] Aunan K, Hansen MH, Liu ZH, Wang SX. The Hidden Hazard of Household Air Pollution in Rural China. Environ Sci Policy. 2019;93:27–33.

[35] Bildirici M. The impacts of governance on environmental pollution in some countries of Middle East and sub-Saharan Africa: the evidence from panel quantile regression and causality. Environ Sci Pollut Res. 2022;29(12):17382–93. doi: 10.1007/s11356-021-15716-2 34665419

[36] Faguet JP. Decentralization and Governance. World Dev. 2013;53:2–13.

[37] Sheng J, Webber M. Incentive-compatible payments for watershed services along the Eastern Route of China’s South-North Water Transfer Project. Ecosys Serv. 2017;25:213–26.

[38] Woodman S. The dynamics of localized citizenship at the grassroots in China. Citizenship Studies. 2022;26(4–5):712–7.

[39] Ruan J, Wang P. Elite Capture and Corruption: The Influence of Elite Collusion on Village Elections and Rural Land Development in China. China Q. 2023;253:107–22.

[40] Poudyal BH, Khatri DB, Paudel D, Marquardt K, Khatri S. Examining forest transition and collective action in Nepal’s community forestry. Land use policy. 2023;134.

[41] Lohmar B, Wang J, Rozelle S, Huang J, Dawe D, States U. China’s Agricultural Water Policy Reforms: Increasing Investment, Resolving Conflicts, and Revising Incentives, Agric. Inf. Bull., 782. 2003.

[42] Cai MN, Sun X. Institutional bindingness, power structure, and land expropriation in China. World Dev. 2018;109:172–86.

[43] Meunier E, Smith P, Griessinger T, Robert C. Understanding changes in reducing pesticide use by farmers: Contribution of the behavioural sciences. Agricultural Systems. 2024;214:103818.

[44] Sumberg J, Okali C. Tomatoes, decentralization, and environmental management in Brong Ahafo, Ghana. Soc Nat Resour. 2006;19(1):19–31.

[45] Liu TT, Bruins RJF, Heberling MT. Factors Influencing Farmers’ Adoption of Best Management Practices: A Review and Synthesis. Sustainability. 2018;10(2). doi: 10.3390/su10020432 29682334 PMC5907504

