# Supplementary material for: Tripartite evolutionary game and simulation analysis of agricultural non-point source pollution control
Source: PLoS One. 2024 Jun 28;19(6):e0305191. doi: 10.1371/journal.pone.0305191 (PMC11213357; doi:10.1371/journal.pone.0305191)
Supplement: S1 File — (PDF) [file pone.0305191.s001.pdf]

Dear Editor:

Thank you for your letter.

Regarding parameter assignment, this paper selects the List of Advanced Counties in the National Village Cleanup Initiative in 2021 (hereinafter referred to as 'the List') published by the Ministry of Agriculture and Rural Development as the initial sample set①. The List recognises 98 advanced counties with excellent performance in ANSPC in 31 provinces, autonomous regions and municipalities directly under the central government; therefore, the advanced counties in the List are representative and typical nationwide. Second, this paper uses a combination of random and stratified sampling, from which 20 advanced counties were selected for data collection and survey. Again, this paper compiles the actual values of documents such as official documents, reports and information on ANSPC parameters of the 20 advanced counties through the Internet and yearbooks. These include, but are not limited to, model farmer profiles, public government reports on financial support, ANSPC costs, fines and revenues. To ensure the accuracy of the data, this paper also uses telephone interviews with demonstration village governments, leaders of village collectives, and experts in related fields. Finally, this paper summarises the actual values of different demonstration farmers and takes the average, scales the parameters equally and derives the numerical assumptions, as shown in Table.

① https://www.moa.gov.cn/govpublic/ncshsycjs/202201/t20220127_6387828.htm

Parameter assignment

ΔG	T_{g1}	T_{g2}	F_v	A	T_{p1}	F_p	B_g	D_g	ΔV	B_{v1}	D_{v1}	T_{p2}	B_{v2}	D_{v2}	α	β	M_{p1}	C_{p1}	C_{p2}
5	6	5	3	6	3	2	1	2	5	3	1	3	1	2	0.5	0.5	2	2.5	5

Fig 1

```
DeltaG=5,Tg1=6,Tg2=5,Fv=1,A=8,Tp1=3,Fp=2,Bg=1,Dg=2,DeltaV=8,Bv1=3,Dv1
=1,Tp2=3,Bv2=1,Dv2=2,alpha=0.5,beta=0.5,Mp1=2,Cp1=4,Cp2=3;

linewidth_line = 0.5;
linewidth_gca = 0.8;
fontsize_gca = 6;
fontsize_label = 8;
fontsize_legend = 6;

for i=0.1:0.2:1
    for j=0.1:0.2:1
        for k=0.1:0.2:1
            [t,y]=ode45(@(t,y)
huanjing(t,y,DeltaG,Tg1,Tg2,Fv,A,Tp1,Fp,Bg,Dg,DeltaV,Bv1,Dv1,Tp2,Bv2,
Dv2,alpha,beta,Mp1,Cp1,Cp2),[0 70],[i j k]);
            figure(1)
            grid on
            plot3(y(:,1),y(:,2),y(:,3),'linewidth',linewidth_line);

set(gca,'XTick',[0:0.2:1],'YTick',[0:0.2:1],'ZTick',[0:0.2:1],'linewi
dth',linewidth_gca,'fontsize',fontsize_gca);
            hold on
            axis([0 1 0 1 0 1])
            view([45 10])
        end
    end
end
xlabel('x','Rotation',0,'fontsize',fontsize_label,'Fontname','Times
New Roman','FontWeight','bold');
ylabel('y','Rotation',0,'fontsize',fontsize_label,'Fontname','Times
New Roman','FontWeight','bold');
zlabel('z','Rotation',360,'position',[0 0
1.05],'fontsize',fontsize_label,'Fontname','Times New
Roman','FontWeight','bold');

hfig = figure(1);
figWidth = 7.99;
figHeight = 5.5;
set(hfig,'PaperUnits','centimeters');
set(hfig,'PaperPosition',[0 0 figWidth figHeight]);
fileout = 'figure 4(a)';
print(hfig,[fileout],'-r500','-dtiff');
```

```
title('FontWeight','bold','position',[1 0 -0.13]);

clear;
DeltaG=5,Tg1=6,Tg2=5,Fv=3,A=6,Tp1=3,Fp=1,Bg=1,Dg=2,DeltaV=5,Bv1=3,Dv1
=1,Tp2=3,Bv2=1,Dv2=2,alpha=0.5,beta=0.5,Mp1=2,Cp1=4,Cp2=3;

linewidth_line = 0.5;
linewidth_gca = 0.8;
fontsize_gca = 6;
fontsize_label = 8;
fontsize_legend = 6;

for i=0.1:0.2:1
    for j=0.1:0.2:1
        for k=0.1:0.2:1
            [t,y]=ode45(@(t,y)
huanjing(t,y,DeltaG,Tg1,Tg2,Fv,A,Tp1,Fp,Bg,Dg,DeltaV,Bv1,Dv1,Tp2,Bv2,
Dv2,alpha,beta,Mp1,Cp1,Cp2),[0 70],[i j k]);
            figure(2)
            grid on
            plot3(y(:,1),y(:,2),y(:,3),'linewidth',linewidth_line);

set(gca,'XTick',[0:0.2:1],'YTick',[0:0.2:1],'ZTick',[0:0.2:1],'linewi
dth',linewidth_gca,'fontsize',fontsize_gca);
            hold on
            axis([0 1 0 1 0 1])
            view([45 10])
        end
    end
end
xlabel('x','Rotation',0,'fontsize',fontsize_label,'Fontname','Times
New Roman','FontWeight','bold');
ylabel('y','Rotation',0,'fontsize',fontsize_label,'Fontname','Times
New Roman','FontWeight','bold');
zlabel('z','Rotation',360,'position',[0 0
1.05],'fontsize',fontsize_label,'Fontname','Times New
Roman','FontWeight','bold');

hfig = figure(2);
figWidth = 7.99;
figHeight = 5.5;
set(hfig,'PaperUnits','centimeters');
set(hfig,'PaperPosition',[0 0 figWidth figHeight]);
fileout = 'figure 4(b)';
```

```
print(hfig,[fileout],'-r500','-dtiff');

clear;
DeltaG=5,Tg1=6,Tg2=5,Fv=3,A=6,Tp1=3,Fp=9,Bg=1,Dg=2,DeltaV=10,Bv1=3,Dv
1=1,Tp2=3,Bv2=1,Dv2=1,alpha=0.5,beta=0.5,Mp1=2,Cp1=1,Cp2=1;

linewidth_line = 0.5;
linewidth_gca = 0.8;
fontsize_gca = 6;
fontsize_label = 8;
fontsize_legend = 6;

for i=0.1:0.2:1
    for j=0.1:0.2:1
        for k=0.1:0.2:1
            [t,y]=ode45(@(t,y)
huanjing(t,y,DeltaG,Tg1,Tg2,Fv,A,Tp1,Fp,Bg,Dg,DeltaV,Bv1,Dv1,Tp2,Bv2,
Dv2,alpha,beta,Mp1,Cp1,Cp2),[0 70],[i j k]);
            figure(3)
            grid on
            plot3(y(:,1),y(:,2),y(:,3),'linewidth',linewidth_line);

set(gca,'XTick',[0:0.2:1],'YTick',[0:0.2:1],'ZTick',[0:0.2:1],'linewi
dth',linewidth_gca,'fontsize',fontsize_gca);
            hold on
            axis([0 1 0 1 0 1])
            view([45 10])
        end
    end
end
xlabel('x','Rotation',0,'fontsize',fontsize_label,'Fontname','Times
New Roman','FontWeight','bold');
ylabel('y','Rotation',0,'fontsize',fontsize_label,'Fontname','Times
New Roman','FontWeight','bold');
zlabel('z','Rotation',360,'position',[0 0
1.05],'fontsize',fontsize_label,'Fontname','Times New
Roman','FontWeight','bold');

hfig = figure(3);
figWidth = 7.99;
figHeight = 5.5;
set(hfig,'PaperUnits','centimeters');
set(hfig,'PaperPosition',[0 0 figWidth figHeight]);
fileout = 'figure 4(c)';
```

```
print(hfig,[fileout],'-r500','-dtiff');

clear;
DeltaG=5,Tg1=8,Tg2=5,Fv=4,A=6,Tp1=0.5,Fp=2,Bg=0,Dg=1,DeltaV=5,Bv1=3,D
v1=1,Tp2=3,Bv2=1,Dv2=2,alpha=0.5,beta=0.5,Mp1=2,Cp1=1,Cp2=3;

linewidth_line = 0.5;
linewidth_gca = 0.8;
fontsize_gca = 6;
fontsize_label = 8;
fontsize_legend = 6;

for i=0.1:0.2:1
    for j=0.1:0.2:1
        for k=0.1:0.2:1
            [t,y]=ode45(@(t,y)
huanjing(t,y,DeltaG,Tg1,Tg2,Fv,A,Tp1,Fp,Bg,Dg,DeltaV,Bv1,Dv1,Tp2,Bv2,
Dv2,alpha,beta,Mp1,Cp1,Cp2),[0 70],[i j k]);
            figure(4)
            grid on
            plot3(y(:,1),y(:,2),y(:,3),'linewidth',linewidth_line);

set(gca,'XTick',[0:0.2:1],'YTick',[0:0.2:1],'ZTick',[0:0.2:1],'linewi
dth',linewidth_gca,'fontsize',fontsize_gca);
            hold on
            axis([0 1 0 1 0 1])
            view([45 10])
        end
    end
end
xlabel('x','Rotation',0,'fontsize',fontsize_label,'Fontname','Times
New Roman','FontWeight','bold');
ylabel('y','Rotation',0,'fontsize',fontsize_label,'Fontname','Times
New Roman','FontWeight','bold');
zlabel('z','Rotation',360,'position',[0 0
1.05],'fontsize',fontsize_label,'Fontname','Times New
Roman','FontWeight','bold');

hfig = figure(4);
figWidth = 7.99;
figHeight = 5.5;
set(hfig,'PaperUnits','centimeters');
set(hfig,'PaperPosition',[0 0 figWidth figHeight]);
fileout = 'figure 4(e)';
```

```
print(hfig,[fileout],'-r500','-dtiff');

clear;
DeltaG=5,Tg1=6,Tg2=5,Fv=3,A=6,Tp1=3,Fp=6,Bg=1,Dg=2,DeltaV=5,Bv1=3,Dv1
=1,Tp2=3,Bv2=1,Dv2=2,alpha=0.5,beta=0.5,Mp1=2,Cp1=2.5,Cp2=5;

linewidth_line = 0.5;
linewidth_gca = 0.8;
fontsize_gca = 6;
fontsize_label = 8;
fontsize_legend = 6;

for i=0.1:0.2:1
    for j=0.1:0.2:1
        for k=0.1:0.2:1
            [t,y]=ode45(@(t,y)
huanjing(t,y,DeltaG,Tg1,Tg2,Fv,A,Tp1,Fp,Bg,Dg,DeltaV,Bv1,Dv1,Tp2,Bv2,
Dv2,alpha,beta,Mp1,Cp1,Cp2),[0 70],[i j k]);
            figure(5)
            grid on
            plot3(y(:,1),y(:,2),y(:,3),'linewidth',linewidth_line);

set(gca,'XTick',[0:0.2:1],'YTick',[0:0.2:1],'ZTick',[0:0.2:1],'linewi
dth',linewidth_gca,'fontsize',fontsize_gca);
            hold on
            axis([0 1 0 1 0 1])
            view([45 10])
        end
    end
end
xlabel('x','Rotation',0,'fontsize',fontsize_label,'Fontname','Times
New Roman','FontWeight','bold');
ylabel('y','Rotation',0,'fontsize',fontsize_label,'Fontname','Times
New Roman','FontWeight','bold');
zlabel('z','Rotation',360,'position',[0 0
1.05],'fontsize',fontsize_label,'Fontname','Times New
Roman','FontWeight','bold');

hfig = figure(5);
figWidth = 7.99;
figHeight = 5.5;
set(hfig,'PaperUnits','centimeters');
set(hfig,'PaperPosition',[0 0 figWidth figHeight]);
fileout = 'figure 4(d)';
```

```
print(hfig,[fileout],'-r500','-dtiff');

clear;
DeltaG=5,Tg1=8,Tg2=5,Fv=4,A=6,Tp1=0.5,Fp=2,Bg=0,Dg=1,DeltaV=5,Bv1=3,D
v1=1,Tp2=3,Bv2=1,Dv2=2,alpha=0.5,beta=0.5,Mp1=2,Cp1=1,Cp2=3;

linewidth_line = 0.5;
linewidth_gca = 0.8;
fontsize_gca = 6;
fontsize_label = 8;
fontsize_legend = 6;

for i=0.1:0.2:1
    for j=0.1:0.2:1
        for k=0.1:0.2:1
            [t,y]=ode45(@(t,y)
huanjing(t,y,DeltaG,Tg1,Tg2,Fv,A,Tp1,Fp,Bg,Dg,DeltaV,Bv1,Dv1,Tp2,Bv2,
Dv2,alpha,beta,Mp1,Cp1,Cp2),[0 70],[i j k]);
            figure(4)
            grid on
            plot3(y(:,1),y(:,2),y(:,3),'linewidth',linewidth_line);

set(gca,'XTick',[0:0.2:1],'YTick',[0:0.2:1],'ZTick',[0:0.2:1],'linewi
dth',linewidth_gca,'fontsize',fontsize_gca);
            hold on
            axis([0 1 0 1 0 1])
            view([45 10])
        end
    end
end
xlabel('x','Rotation',0,'fontsize',fontsize_label,'Fontname','Times
New Roman','FontWeight','bold');
ylabel('y','Rotation',0,'fontsize',fontsize_label,'Fontname','Times
New Roman','FontWeight','bold');
zlabel('z','Rotation',360,'position',[0 0
1.05],'fontsize',fontsize_label,'Fontname','Times New
Roman','FontWeight','bold');

hfig = figure(4);
figWidth = 7.99;
figHeight = 5.5;
set(hfig,'PaperUnits','centimeters');
set(hfig,'PaperPosition',[0 0 figWidth figHeight]);
fileout = 'figure 4(e)';
```

```
print(hfig,[fileout],'-r500','-dtiff');
```

Fig 2

```
clc;clear;
linewidth_line = 0.5;
linewidth_gca = 0.8;
fontsize_gca = 6;
fontsize_label = 8;
fontsize_legend = 4;

DeltaG=5,Tg1=6,Tg2=5,Fv=3,A=6,Tp1=3,Fp=2,Bg=1,Dg=2,DeltaV=5,Bv1=3,Dv1
=1,Tp2=3,Bv2=1,Dv2=2,alpha=0.5,beta=0.5,Mp1=2,Cp1=2.5,Cp2=5;
[t,y]=ode45(@(t,y)
huanjing(t,y,DeltaG,Tg1,Tg2,Fv,A,Tp1,Fp,Bg,Dg,DeltaV,Bv1,Dv1,Tp2,Bv2,
Dv2,alpha,beta,Mp1,Cp1,Cp2),[0 10],[0.5,0.3,0.3]);
figure(1)
grid on
set(gca,'linewidth',linewidth_gca,'fontsize',fontsize_gca);
plot(t,y(:,1),'k-','lineWidth',linewidth_line,'markerfacecolor','k');
hold on
plot(t,y(:,2),'k--
','lineWidth',linewidth_line,'markerfacecolor','k');
hold on
plot(t,y(:,3),'k:','lineWidth',linewidth_line,'markerfacecolor','k');
hold on
set(gca,'XTick',[0:1:10],'YTick',[0:0.1:1],'linewidth',linewidth_gca,
'fontsize',fontsize_gca)
axis([0 10 0 1])
xlabel('Time',
'interpreter','latex','fontsize',fontsize_label,'Fontweight','bold','
fontname','Times New Roman');
ylabel('The probability of strategy
selection','interpreter','latex','fontsize',fontsize_label,'Fontweigh
t','bold','fontname','Times New Roman');
h = legend('Government', 'Village Collective', 'Farmer');
set(h,'fontsize',fontsize_legend,'location','Best')

hfig = figure(1);
figWidth = 7.99;
figHeight = 5.5;
set(hfig,'PaperUnits','centimeters');
set(hfig,'PaperPosition',[0 0 figWidth figHeight]);
fileout = 'figure 5(a)';
print(hfig,[fileout],'-r500','-dtiff');
```

```
clc;clear;
linewidth_line = 0.5;
linewidth_gca = 0.8;
fontsize_gca = 6;
fontsize_label = 8;
fontsize_legend = 4;

DeltaG=5,Tg1=6,Tg2=5,Fv=3,A=6,Tp1=3,Fp=2,Bg=1,Dg=2,DeltaV=5,Bv1=3,Dv1
=1,Tp2=3,Bv2=1,Dv2=2,alpha=0.5,beta=0.5,Mp1=2,Cp1=2.5,Cp2=5;
[t,y]=ode45(@(t,y)
huanjing(t,y,DeltaG,Tg1,Tg2,Fv,A,Tp1,Fp,Bg,Dg,DeltaV,Bv1,Dv1,Tp2,Bv2,
Dv2,alpha,beta,Mp1,Cp1,Cp2),[0 10],[0.8,0.3,0.3]);
figure(2)
grid on
set(gca,'linewidth',linewidth_gca,'fontsize',fontsize_gca);
plot(t,y(:,1),'k-','lineWidth',linewidth_line,'markerfacecolor','k');
hold on
plot(t,y(:,2),'k--
','lineWidth',linewidth_line,'markerfacecolor','k');
hold on
plot(t,y(:,3),'k:','lineWidth',linewidth_line,'markerfacecolor','k');
hold on
set(gca,'XTick',[0:1:10],'YTick',[0:0.1:1],'linewidth',linewidth_gca,
'fontsize',fontsize_gca)
axis([0 10 0 1])
xlabel('Time',
'interpreter','latex','fontsize',fontsize_label,'Fontweight','bold','
fontname','Times New Roman');
ylabel('The probability of strategy
selection','interpreter','latex','fontsize',fontsize_label,'Fontweigh
t','bold','fontname','Times New Roman');
h = legend('Government', 'Village Collective', 'Farmer');
set(h,'fontsize',fontsize_legend,'location','Best')

hfig = figure(2);
figWidth = 7.99;
figHeight = 5.5;
set(hfig,'PaperUnits','centimeters');
set(hfig,'PaperPosition',[0 0 figWidth figHeight]);
fileout = 'figure 5(b)';
print(hfig,[fileout],'-r500','-dtiff');
```

```
clc;clear;
linewidth_line = 0.5;
linewidth_gca = 0.8;
fontsize_gca = 6;
fontsize_label = 8;
fontsize_legend = 4;

DeltaG=5,Tg1=6,Tg2=5,Fv=3,A=6,Tp1=3,Fp=2,Bg=1,Dg=2,DeltaV=5,Bv1=3,Dv1
=1,Tp2=3,Bv2=1,Dv2=2,alpha=0.5,beta=0.5,Mp1=2,Cp1=2.5,Cp2=5;
[t,y]=ode45(@(t,y)
huanjing(t,y,DeltaG,Tg1,Tg2,Fv,A,Tp1,Fp,Bg,Dg,DeltaV,Bv1,Dv1,Tp2,Bv2,
Dv2,alpha,beta,Mp1,Cp1,Cp2),[0 10],[0.5,0.5,0.3]);
figure(3)
grid on
set(gca,'linewidth',linewidth_gca,'fontsize',fontsize_gca);
plot(t,y(:,1),'k-','lineWidth',linewidth_line,'markerfacecolor','k');
hold on
plot(t,y(:,2),'k--
','lineWidth',linewidth_line,'markerfacecolor','k');
hold on
plot(t,y(:,3),'k:','lineWidth',linewidth_line,'markerfacecolor','k');
hold on
set(gca,'XTick',[0:1:10],'YTick',[0:0.1:1],'linewidth',linewidth_gca,
'fontsize',fontsize_gca)
axis([0 10 0 1])
xlabel('Time',
'interpreter','latex','fontsize',fontsize_label,'Fontweight','bold','
fontname','Times New Roman');
ylabel('The probability of strategy
selection','interpreter','latex','fontsize',fontsize_label,'Fontweigh
t','bold','fontname','Times New Roman');
h = legend('Government', 'Village Collective', 'Farmer');
set(h,'fontsize',fontsize_legend,'location','Best')

hfig = figure(3);
figWidth = 7.99;
figHeight = 5.5;
set(hfig,'PaperUnits','centimeters');
set(hfig,'PaperPosition',[0 0 figWidth figHeight]);
fileout = 'figure 5(c)';
print(hfig,[fileout],'-r500','-dtiff');
```

```
clc;clear;
linewidth_line = 0.5;
linewidth_gca = 0.8;
fontsize_gca = 6;
fontsize_label = 8;
fontsize_legend = 4;

DeltaG=5,Tg1=6,Tg2=5,Fv=3,A=6,Tp1=3,Fp=2,Bg=1,Dg=2,DeltaV=5,Bv1=3,Dv1
=1,Tp2=3,Bv2=1,Dv2=2,alpha=0.5,beta=0.5,Mp1=2,Cp1=2.5,Cp2=5;
[t,y]=ode45(@(t,y)
huanjing(t,y,DeltaG,Tg1,Tg2,Fv,A,Tp1,Fp,Bg,Dg,DeltaV,Bv1,Dv1,Tp2,Bv2,
Dv2,alpha,beta,Mp1,Cp1,Cp2),[0 10],[0.5,0.3,0.5]);
figure(4)
grid on
set(gca,'linewidth',linewidth_gca,'fontsize',fontsize_gca);
plot(t,y(:,1),'k-','lineWidth',linewidth_line,'markerfacecolor','k');
hold on
plot(t,y(:,2),'k--
','lineWidth',linewidth_line,'markerfacecolor','k');
hold on
plot(t,y(:,3),'k:','lineWidth',linewidth_line,'markerfacecolor','k');
hold on
set(gca,'XTick',[0:1:10],'YTick',[0:0.1:1],'linewidth',linewidth_gca,
'fontsize',fontsize_gca)
axis([0 10 0 1])
xlabel('Time',
'interpreter','latex','fontsize',fontsize_label,'Fontweight','bold','
fontname','Times New Roman');
ylabel('The probability of strategy
selection','interpreter','latex','fontsize',fontsize_label,'Fontweigh
t','bold','fontname','Times New Roman');
h = legend('Government', 'Village Collective', 'Farmer');
set(h,'fontsize',fontsize_legend,'location','Best')

hfig = figure(4);
figWidth = 7.99;
figHeight = 5.5;
set(hfig,'PaperUnits','centimeters');
set(hfig,'PaperPosition',[0 0 figWidth figHeight]);
fileout = 'figure 5(d)';
print(hfig,[fileout],'-r500','-dtiff');
```

Fig 3

```
clc;clear;
linewidth_line = 0.5;
linewidth_gca = 0.8;
fontsize_gca = 6;
fontsize_label = 8;
fontsize_legend = 4;
DeltaG=5,Tg1=6,Tg2=5,Fv=3,Tp1=3,Fp=2,Bg=1,Dg=2,DeltaV=5,Dv1=1,Tp2=3,B
v2=1,Dv2=2,alpha=0.5,beta=0.5,Mp1=2,Cp1=2.5,Cp2=5,Bv1=3;
figure(1)
for A=6;
    [t,y]=ode45(@(t,y)
huanjing(t,y,DeltaG,Tg1,Tg2,Fv,A,Tp1,Fp,Bg,Dg,DeltaV,Bv1,Dv1,Tp2,Bv2,
Dv2,alpha,beta,Mp1,Cp1,Cp2),[0 10],[0.5,0.5,0.5]);
   plot(t,y(:,1),'k-
','lineWidth',linewidth_line,'markerfacecolor','k');
   hold on
end
for A=8;
    [t,y]=ode45(@(t,y)
huanjing(t,y,DeltaG,Tg1,Tg2,Fv,A,Tp1,Fp,Bg,Dg,DeltaV,Bv1,Dv1,Tp2,Bv2,
Dv2,alpha,beta,Mp1,Cp1,Cp2),[0 10],[0.5,0.5,0.5]);
   plot(t,y(:,1),'k--
','lineWidth',linewidth_line,'markerfacecolor','k');
   hold on
end
for A=10;
    [t,y]=ode45(@(t,y)
huanjing(t,y,DeltaG,Tg1,Tg2,Fv,A,Tp1,Fp,Bg,Dg,DeltaV,Bv1,Dv1,Tp2,Bv2,
Dv2,alpha,beta,Mp1,Cp1,Cp2),[0 10],[0.5,0.5,0.5]);

plot(t,y(:,1),'k:','lineWidth',linewidth_line,'markerfacecolor','k');
   hold on
end
set(gca,'XTick',[0:1:10],'YTick',[0:0.1:1],'linewidth',linewidth_gca,
'fontsize',fontsize_gca)
axis([0 10 0 1])
xlabel('Time',
'interpreter','latex','fontsize',fontsize_label,'Fontweight','bold','
fontname','Times New Roman');
ylabel('The probability of strategy
selection','interpreter','latex','fontsize',fontsize_label,'Fontweigh
```

```
t','bold','fontname','Times New Roman');
h = legend('A=6 ', 'A=8 ', 'A=10 ');
set(h,'fontsize',fontsize_legend,'location','Best')

hfig = figure(1);
figWidth = 7.99;
figHeight = 5.5;
set(hfig,'PaperUnits','centimeters');
set(hfig,'PaperPosition',[0 0 figWidth figHeight]);
fileout = 'figure 6(a)';
print(hfig,[fileout],'-r500','-dtiff');

clc;clear;
linewidth_line = 0.5;
linewidth_gca = 0.8;
fontsize_gca = 6;
fontsize_label = 8;
fontsize_legend = 4;
DeltaG=5,Tg1=6,Tg2=5,Fv=3,Tp1=3,Fp=2,Bg=1,Dg=2,DeltaV=5,Dv1=1,Tp2=3,B
v2=1,Dv2=2,alpha=0.5,beta=0.5,Mp1=2,Cp1=2.5,Cp2=5,Bv1=3;
figure(2)
for A=6;
    [t,y]=ode45(@(t,y)
huanjing(t,y,DeltaG,Tg1,Tg2,Fv,A,Tp1,Fp,Bg,Dg,DeltaV,Bv1,Dv1,Tp2,Bv2,
Dv2,alpha,beta,Mp1,Cp1,Cp2),[0 10],[0.5,0.5,0.5]);
    plot(t,y(:,2),'k-
','lineWidth',linewidth_line,'markerfacecolor','k');
    hold on
end
for A=8;
    [t,y]=ode45(@(t,y)
huanjing(t,y,DeltaG,Tg1,Tg2,Fv,A,Tp1,Fp,Bg,Dg,DeltaV,Bv1,Dv1,Tp2,Bv2,
Dv2,alpha,beta,Mp1,Cp1,Cp2),[0 10],[0.5,0.5,0.5]);
    plot(t,y(:,2),'k--
','lineWidth',linewidth_line,'markerfacecolor','k');
    hold on
end
for A=10;
    [t,y]=ode45(@(t,y)
huanjing(t,y,DeltaG,Tg1,Tg2,Fv,A,Tp1,Fp,Bg,Dg,DeltaV,Bv1,Dv1,Tp2,Bv2,
Dv2,alpha,beta,Mp1,Cp1,Cp2),[0 10],[0.5,0.5,0.5]);

plot(t,y(:,2),'k:','lineWidth',linewidth_line,'markerfacecolor','k');
    hold on
```

```
end
set(gca,'XTick',[0:1:10],'YTick',[0:0.1:1],'linewidth',linewidth_gca,
'fontsize',fontsize_gca)
axis([0 10 0 1])
xlabel('Time',
'interpreter','latex','fontsize',fontsize_label,'Fontweight','bold','
fontname','Times New Roman');
ylabel('The probability of strategy
selection','interpreter','latex','fontsize',fontsize_label,'Fontweigh
t','bold','fontname','Times New Roman');
h = legend('A=6 ', 'A=8 ', 'A=10 ');
set(h,'fontsize',fontsize_legend,'location','Best')

hfig = figure(2);
figWidth = 7.99;
figHeight = 5.5;
set(hfig,'PaperUnits','centimeters');
set(hfig,'PaperPosition',[0 0 figWidth figHeight]);
fileout = 'figure 6(b)';
print(hfig,[fileout],'-r500','-dtiff');

clc;clear;
linewidth_line = 0.5;
linewidth_gca = 0.8;
fontsize_gca = 6;
fontsize_label = 8;
fontsize_legend = 4;
DeltaG=5,Tg1=6,Tg2=5,Fv=3,Tp1=3,Fp=2,Bg=1,Dg=2,DeltaV=5,Dv1=1,Tp2=3,B
v2=1,Dv2=2,alpha=0.5,beta=0.5,Mp1=2,Cp1=2.5,Cp2=5,Bv1=3;
figure(3)
for A=6;
    [t,y]=ode45(@(t,y)
huanjing(t,y,DeltaG,Tg1,Tg2,Fv,A,Tp1,Fp,Bg,Dg,DeltaV,Bv1,Dv1,Tp2,Bv2,
Dv2,alpha,beta,Mp1,Cp1,Cp2),[0 10],[0.5,0.5,0.5]);
    plot(t,y(:,3),'k-
','lineWidth',linewidth_line,'markerfacecolor','k');
    hold on
end
for A=8;
    [t,y]=ode45(@(t,y)
huanjing(t,y,DeltaG,Tg1,Tg2,Fv,A,Tp1,Fp,Bg,Dg,DeltaV,Bv1,Dv1,Tp2,Bv2,
Dv2,alpha,beta,Mp1,Cp1,Cp2),[0 10],[0.5,0.5,0.5]);
    plot(t,y(:,3),'k--
','lineWidth',linewidth_line,'markerfacecolor','k');
```

```
    hold on
end
for A=10;
    [t,y]=ode45(@(t,y)
huanjing(t,y,DeltaG,Tg1,Tg2,Fv,A,Tp1,Fp,Bg,Dg,DeltaV,Bv1,Dv1,Tp2,Bv2,
Dv2,alpha,beta,Mp1,Cp1,Cp2),[0 10],[0.5,0.5,0.5]);

plot(t,y(:,1),'k:','lineWidth',linewidth_line,'markerfacecolor','k');
    hold on
end
set(gca,'XTick',[0:1:10],'YTick',[0:0.1:1],'linewidth',linewidth_gca,
'fontsize',fontsize_gca)
axis([0 10 0 1])
xlabel('Time',
'interpreter','latex','fontsize',fontsize_label,'Fontweight','bold','
fontname','Times New Roman');
ylabel('The probability of strategy
selection','interpreter','latex','fontsize',fontsize_label,'Fontweigh
t','bold','fontname','Times New Roman');
h = legend('A=6 ', 'A=8 ', 'A=10 ');
set(h,'fontsize',fontsize_legend,'location','Best')

hfig = figure(3);
figWidth = 7.99;
figHeight = 5.5;
set(hfig,'PaperUnits','centimeters');
set(hfig,'PaperPosition',[0 0 figWidth figHeight]);
fileout = 'figure 6(c)';
print(hfig,[fileout],'-r500','-dtiff');
```

Fig 4

```
clc;clear;
linewidth_line = 0.5;
linewidth_gca = 0.8;
fontsize_gca = 6;
fontsize_label = 8;
fontsize_legend = 4;
DeltaG=5,Tg1=6,Tg2=5,A=6,Tp1=3,Fp=2,Bg=1,Dg=2,DeltaV=5,Bv1=3,Dv1=1,Tp
2=3,Bv2=1,Dv2=2,alpha=0.5,beta=0.5,Mp1=2,Cp1=2.5,Cp2=5;
figure(1)
for Fv=1.5;
    [t,y]=ode45(@(t,y)
huanjing(t,y,DeltaG,Tg1,Tg2,Fv,A,Tp1,Fp,Bg,Dg,DeltaV,Bv1,Dv1,Tp2,Bv2,
Dv2,alpha,beta,Mp1,Cp1,Cp2),[0 10],[0.5,0.5,0.5]);
```

```
    plot(t,y(:,1),'k-
','lineWidth',linewidth_line,'markerfacecolor','k');
    hold on
end
for Fv=3;
    [t,y]=ode45(@(t,y)
huanjing(t,y,DeltaG,Tg1,Tg2,Fv,A,Tp1,Fp,Bg,Dg,DeltaV,Bv1,Dv1,Tp2,Bv2,
Dv2,alpha,beta,Mp1,Cp1,Cp2),[0 10],[0.5,0.5,0.5]);
    plot(t,y(:,1),'k--
','lineWidth',linewidth_line,'markerfacecolor','k');
    hold on
end
for Fv=4.5;
    [t,y]=ode45(@(t,y)
huanjing(t,y,DeltaG,Tg1,Tg2,Fv,A,Tp1,Fp,Bg,Dg,DeltaV,Bv1,Dv1,Tp2,Bv2,
Dv2,alpha,beta,Mp1,Cp1,Cp2),[0 10],[0.5,0.5,0.5]);

plot(t,y(:,1),'k:','lineWidth',linewidth_line,'markerfacecolor','k');
    hold on
end
set(gca,'XTick',[0:1:10],'YTick',[0:0.1:1],'linewidth',linewidth_gca,
'fontsize',fontsize_gca)
axis([0 10 0 1])
xlabel('Time',
'interpreter','latex','fontsize',fontsize_label,'Fontweight','bold','
fontname','Times New Roman');
ylabel('The probability of strategy
selection','interpreter','latex','fontsize',fontsize_label,'Fontweigh
t','bold','fontname','Times New Roman');
h = legend('Fv=1.5 ', 'Fv=3 ', 'Fv=4.5');
set(h,'fontsize',fontsize_legend,'location','Best')

hfig = figure(1);
figWidth = 7.99;
figHeight = 5.5;
set(hfig,'PaperUnits','centimeters');
set(hfig,'PaperPosition',[0 0 figWidth figHeight]);
fileout = 'figure 7(a)';
print(hfig,[fileout],'-r500','-dtiff');

clc;clear;
linewidth_line = 0.5;
linewidth_gca = 0.8;
fontsize_gca = 6;
```

```
fontsize_label = 8;
fontsize_legend = 4;
DeltaG=5,Tg1=6,Tg2=5,A=6,Tp1=3,Fp=2,Bg=1,Dg=2,DeltaV=5,Bv1=3,Dv1=1,Tp
2=3,Bv2=1,Dv2=2,alpha=0.5,beta=0.5,Mp1=2,Cp1=2.5,Cp2=5;
figure(2)
for Fv=1.5;
    [t,y]=ode45(@(t,y)
huanjing(t,y,DeltaG,Tg1,Tg2,Fv,A,Tp1,Fp,Bg,Dg,DeltaV,Bv1,Dv1,Tp2,Bv2,
Dv2,alpha,beta,Mp1,Cp1,Cp2),[0 10],[0.5,0.5,0.5]);
    plot(t,y(:,2),'k-
','lineWidth',linewidth_line,'markerfacecolor','k');
    hold on
end
for Fv=3;
    [t,y]=ode45(@(t,y)
huanjing(t,y,DeltaG,Tg1,Tg2,Fv,A,Tp1,Fp,Bg,Dg,DeltaV,Bv1,Dv1,Tp2,Bv2,
Dv2,alpha,beta,Mp1,Cp1,Cp2),[0 10],[0.5,0.5,0.5]);
    plot(t,y(:,2),'k--
','lineWidth',linewidth_line,'markerfacecolor','k');
    hold on
end
for Fv=4.5;
    [t,y]=ode45(@(t,y)
huanjing(t,y,DeltaG,Tg1,Tg2,Fv,A,Tp1,Fp,Bg,Dg,DeltaV,Bv1,Dv1,Tp2,Bv2,
Dv2,alpha,beta,Mp1,Cp1,Cp2),[0 10],[0.5,0.5,0.5]);

plot(t,y(:,2),'k:','lineWidth',linewidth_line,'markerfacecolor','k');
    hold on
end
set(gca,'XTick',[0:1:10],'YTick',[0:0.1:1],'linewidth',linewidth_gca,
'fontsize',fontsize_gca)
axis([0 10 0 1])
xlabel('Time',
'interpreter','latex','fontsize',fontsize_label,'Fontweight','bold','
fontname','Times New Roman');
ylabel('The probability of strategy
selection','interpreter','latex','fontsize',fontsize_label,'Fontweigh
t','bold','fontname','Times New Roman');
h = legend('Fv=1.5 ', 'Fv=3 ', 'Fv=4.5');
set(h,'fontsize',fontsize_legend,'location','Best')

hfig = figure(2);
figWidth = 7.99;
figHeight = 5.5;
```

```
set(hfig,'PaperUnits','centimeters');
set(hfig,'PaperPosition',[0 0 figWidth figHeight]);
fileout = 'figure 7(b)';
print(hfig,[fileout],'-r500','-dtiff');

clc;clear;
linewidth_line = 0.5;
linewidth_gca = 0.8;
fontsize_gca = 6;
fontsize_label = 8;
fontsize_legend = 4;
DeltaG=5,Tg1=6,Tg2=5,A=6,Tp1=3,Fp=2,Bg=1,Dg=2,DeltaV=5,Bv1=3,Dv1=1,Tp
2=3,Bv2=1,Dv2=2,alpha=0.5,beta=0.5,Mp1=2,Cp1=2.5,Cp2=5;
figure(3)
for Fv=1.5;
    [t,y]=ode45(@(t,y)
huanjing(t,y,DeltaG,Tg1,Tg2,Fv,A,Tp1,Fp,Bg,Dg,DeltaV,Bv1,Dv1,Tp2,Bv2,
Dv2,alpha,beta,Mp1,Cp1,Cp2),[0 10],[0.5,0.5,0.5]);
    plot(t,y(:,3),'k-
','lineWidth',linewidth_line,'markerfacecolor','k');
    hold on
end
for Fv=3;
    [t,y]=ode45(@(t,y)
huanjing(t,y,DeltaG,Tg1,Tg2,Fv,A,Tp1,Fp,Bg,Dg,DeltaV,Bv1,Dv1,Tp2,Bv2,
Dv2,alpha,beta,Mp1,Cp1,Cp2),[0 10],[0.5,0.5,0.5]);
    plot(t,y(:,3),'k--
','lineWidth',linewidth_line,'markerfacecolor','k');
    hold on
end
for Fv=4.5;
    [t,y]=ode45(@(t,y)
huanjing(t,y,DeltaG,Tg1,Tg2,Fv,A,Tp1,Fp,Bg,Dg,DeltaV,Bv1,Dv1,Tp2,Bv2,
Dv2,alpha,beta,Mp1,Cp1,Cp2),[0 10],[0.5,0.5,0.5]);

plot(t,y(:,3),'k:','lineWidth',linewidth_line,'markerfacecolor','k');
    hold on
end
set(gca,'XTick',[0:1:10],'YTick',[0:0.1:1],'linewidth',linewidth_gca,
'fontsize',fontsize_gca)
axis([0 10 0 1])
xlabel('Time',
'interpreter','latex','fontsize',fontsize_label,'Fontweight','bold','
fontname','Times New Roman');
```

```
ylabel('The probability of strategy selection','interpreter','latex','fontsize',fontsize_label,'Fontweight','bold','fontname','Times New Roman');
h = legend('Fv=1.5 ', 'Fv=3 ', 'Fv=4.5');
set(h,'fontsize',fontsize_legend,'location','Best')

hfig = figure(3);
figWidth = 7.99;
figHeight = 5.5;
set(hfig,'PaperUnits','centimeters');
set(hfig,'PaperPosition',[0 0 figWidth figHeight]);
fileout = 'figure 7(c)';
print(hfig,[fileout],'-r500','-dtiff');

clc;clear;
linewidth_line = 0.5;
linewidth_gca = 0.8;
fontsize_gca = 6;
fontsize_label = 8;
fontsize_legend = 4;
DeltaG=5,Tg1=6,Tg2=5,Fv=3,A=6,Tp1=3,Bg=1,Dg=2,DeltaV=5,Bv1=3,Dv1=1,Tp2=3,Bv2=1,Dv2=2,alpha=0.5,beta=0.5,Mp1=2,Cp1=2.5,Cp2=5;
figure(1)
for Fp=1.2;
    [t,y]=ode45(@(t,y) huanjing(t,y,DeltaG,Tg1,Tg2,Fv,A,Tp1,Fp,Bg,Dg,DeltaV,Bv1,Dv1,Tp2,Bv2,Dv2,alpha,beta,Mp1,Cp1,Cp2),[0 10],[0.5,0.5,0.5]);
    plot(t,y(:,1),'k-','lineWidth',linewidth_line,'markerfacecolor','k');
    hold on
end
for Fp=2;
    [t,y]=ode45(@(t,y) huanjing(t,y,DeltaG,Tg1,Tg2,Fv,A,Tp1,Fp,Bg,Dg,DeltaV,Bv1,Dv1,Tp2,Bv2,Dv2,alpha,beta,Mp1,Cp1,Cp2),[0 10],[0.5,0.5,0.5]);
    plot(t,y(:,1),'k--','lineWidth',linewidth_line,'markerfacecolor','k');
    hold on
end
for Fp=2.8;
    [t,y]=ode45(@(t,y) huanjing(t,y,DeltaG,Tg1,Tg2,Fv,A,Tp1,Fp,Bg,Dg,DeltaV,Bv1,Dv1,Tp2,Bv2,Dv2,alpha,beta,Mp1,Cp1,Cp2),[0 10],[0.5,0.5,0.5]);
```

```
plot(t,y(:,1),'k:','lineWidth',linewidth_line,'markerfacecolor','k');
    hold on
end
set(gca,'XTick',[0:1:10],'YTick',[0:0.1:1],'linewidth',linewidth_gca,
'fontsize',fontsize_gca)
axis([0 10 0 1])
xlabel('Time',
'interpreter','latex','fontsize',fontsize_label,'Fontweight','bold','
fontname','Times New Roman');
ylabel('The probability of strategy
selection','interpreter','latex','fontsize',fontsize_label,'Fontweigh
t','bold','fontname','Times New Roman');
h = legend('Fp=1.2 ', 'Fp=2 ', 'Fp=2.8');
set(h,'fontsize',fontsize_legend,'location','Best')

hfig = figure(1);
figWidth = 7.99;
figHeight = 5.5;
set(hfig,'PaperUnits','centimeters');
set(hfig,'PaperPosition',[0 0 figWidth figHeight]);
fileout = 'figure 7(d)';
print(hfig,[fileout],'-r500','-dtiff');

clc;clear;
linewidth_line = 0.5;
linewidth_gca = 0.8;
fontsize_gca = 6;
fontsize_label = 8;
fontsize_legend = 4;
DeltaG=5,Tg1=6,Tg2=5,Fv=3,A=6,Tp1=3,Bg=1,Dg=2,DeltaV=5,Bv1=3,Dv1=1,Tp
2=3,Bv2=1,Dv2=2,alpha=0.5,beta=0.5,Mp1=2,Cp1=2.5,Cp2=5;
figure(2)
for Fp=1.2;
    [t,y]=ode45(@(t,y)
huanjing(t,y,DeltaG,Tg1,Tg2,Fv,A,Tp1,Fp,Bg,Dg,DeltaV,Bv1,Dv1,Tp2,Bv2,
Dv2,alpha,beta,Mp1,Cp1,Cp2),[0 10],[0.5,0.5,0.5]);
    plot(t,y(:,2),'k-
','lineWidth',linewidth_line,'markerfacecolor','k');
    hold on
end
for Fp=2;
    [t,y]=ode45(@(t,y)
huanjing(t,y,DeltaG,Tg1,Tg2,Fv,A,Tp1,Fp,Bg,Dg,DeltaV,Bv1,Dv1,Tp2,Bv2,
Dv2,alpha,beta,Mp1,Cp1,Cp2),[0 10],[0.5,0.5,0.5]);
```

```
    plot(t,y(:,2),'k--
','lineWidth',linewidth_line,'markerfacecolor','k');
    hold on
end
for Fp=2.8;
    [t,y]=ode45(@(t,y)
huanjing(t,y,DeltaG,Tg1,Tg2,Fv,A,Tp1,Fp,Bg,Dg,DeltaV,Bv1,Dv1,Tp2,Bv2,
Dv2,alpha,beta,Mp1,Cp1,Cp2),[0 10],[0.5,0.5,0.5]);

plot(t,y(:,2),'k:','lineWidth',linewidth_line,'markerfacecolor','k');
    hold on
end
set(gca,'XTick',[0:1:10],'YTick',[0:0.1:1],'linewidth',linewidth_gca,
'fontsize',fontsize_gca)
axis([0 10 0 1])
xlabel('Time',
'interpreter','latex','fontsize',fontsize_label,'Fontweight','bold','
fontname','Times New Roman');
ylabel('The probability of strategy
selection','interpreter','latex','fontsize',fontsize_label,'Fontweigh
t','bold','fontname','Times New Roman');
h = legend('Fp=1.2 ', 'Fp=2 ', 'Fp=2.8');
set(h,'fontsize',fontsize_legend,'location','Best')

hfig = figure(2);
figWidth = 7.99;
figHeight = 5.5;
set(hfig,'PaperUnits','centimeters');
set(hfig,'PaperPosition',[0 0 figWidth figHeight]);
fileout = 'figure 7(e)';
print(hfig,[fileout],'-r500','-dtiff');

clc;clear;
linewidth_line = 0.5;
linewidth_gca = 0.8;
fontsize_gca = 6;
fontsize_label = 8;
fontsize_legend = 4;
DeltaG=5,Tg1=6,Tg2=5,Fv=3,A=6,Tp1=3,Bg=1,Dg=2,DeltaV=5,Bv1=3,Dv1=1,Tp
2=3,Bv2=1,Dv2=2,alpha=0.5,beta=0.5,Mp1=2,Cp1=2.5,Cp2=5;
figure(3)
for Fp=1.2;
    [t,y]=ode45(@(t,y)
huanjing(t,y,DeltaG,Tg1,Tg2,Fv,A,Tp1,Fp,Bg,Dg,DeltaV,Bv1,Dv1,Tp2,Bv2,
```

```
Dv2,alpha,beta,Mp1,Cp1,Cp2),[0 10],[0.5,0.5,0.5]);
    plot(t,y(:,3),'k-
','lineWidth',linewidth_line,'markerfacecolor','k');
    hold on
end
for Fp=2;
    [t,y]=ode45(@(t,y)
huanjing(t,y,DeltaG,Tg1,Tg2,Fv,A,Tp1,Fp,Bg,Dg,DeltaV,Bv1,Dv1,Tp2,Bv2,
Dv2,alpha,beta,Mp1,Cp1,Cp2),[0 10],[0.5,0.5,0.5]);
    plot(t,y(:,3),'k--
','lineWidth',linewidth_line,'markerfacecolor','k');
    hold on
end
for Fp=2.8;
    [t,y]=ode45(@(t,y)
huanjing(t,y,DeltaG,Tg1,Tg2,Fv,A,Tp1,Fp,Bg,Dg,DeltaV,Bv1,Dv1,Tp2,Bv2,
Dv2,alpha,beta,Mp1,Cp1,Cp2),[0 10],[0.5,0.5,0.5]);

plot(t,y(:,3),'k:','lineWidth',linewidth_line,'markerfacecolor','k');
    hold on
end
set(gca,'XTick',[0:1:10],'YTick',[0:0.1:1],'linewidth',linewidth_gca,
'fontsize',fontsize_gca)
axis([0 10 0 1])
xlabel('Time',
'interpreter','latex','fontsize',fontsize_label,'Fontweight','bold','
fontname','Times New Roman');
ylabel('The probability of strategy
selection','interpreter','latex','fontsize',fontsize_label,'Fontweigh
t','bold','fontname','Times New Roman');
h = legend('Fp=1.2 ', 'Fp=2 ', 'Fp=2.8');
set(h,'fontsize',fontsize_legend,'location','Best')

hfig = figure(3);
figWidth = 7.99;
figHeight = 5.5;
set(hfig,'PaperUnits','centimeters');
set(hfig,'PaperPosition',[0 0 figWidth figHeight]);
fileout = 'figure 7(f)';
print(hfig,[fileout],'-r500','-dtiff');
```

Fig 5

```
clc;clear;
linewidth_line = 0.5;
```

```
linewidth_gca = 0.8;
fontsize_gca = 6;
fontsize_label = 8;
fontsize_legend = 4;
DeltaG=5,Tg1=6,Tg2=5,Fv=3,A=6,Tp1=3,Fp=2,Bg=1,Dg=2,DeltaV=5,Dv1=1,Tp2
=3,Bv2=1,Dv2=2,alpha=0.5,beta=0.5,Mp1=2,Cp1=2.5,Cp2=5;
figure(1)
for Bv1=3;
    [t,y]=ode45(@(t,y)
huanjing(t,y,DeltaG,Tg1,Tg2,Fv,A,Tp1,Fp,Bg,Dg,DeltaV,Bv1,Dv1,Tp2,Bv2,
Dv2,alpha,beta,Mp1,Cp1,Cp2),[0 10],[0.5,0.5,0.5]);
    plot(t,y(:,1),'k-
','lineWidth',linewidth_line,'markerfacecolor','k');
    hold on
end
for Bv1=6;
    [t,y]=ode45(@(t,y)
huanjing(t,y,DeltaG,Tg1,Tg2,Fv,A,Tp1,Fp,Bg,Dg,DeltaV,Bv1,Dv1,Tp2,Bv2,
Dv2,alpha,beta,Mp1,Cp1,Cp2),[0 10],[0.5,0.5,0.5]);
    plot(t,y(:,1),'k--
','lineWidth',linewidth_line,'markerfacecolor','k');
    hold on
end
for Bv1=9;
    [t,y]=ode45(@(t,y)
huanjing(t,y,DeltaG,Tg1,Tg2,Fv,A,Tp1,Fp,Bg,Dg,DeltaV,Bv1,Dv1,Tp2,Bv2,
Dv2,alpha,beta,Mp1,Cp1,Cp2),[0 10],[0.5,0.5,0.5]);

plot(t,y(:,1),'k:','lineWidth',linewidth_line,'markerfacecolor','k');
    hold on
end
set(gca,'XTick',[0:1:10],'YTick',[0:0.1:1],'linewidth',linewidth_gca,
'fontsize',fontsize_gca)
axis([0 10 0 1])
xlabel('Time',
'interpreter','latex','fontsize',fontsize_label,'Fontweight','bold','
fontname','Times New Roman');
ylabel('The probability of strategy
selection','interpreter','latex','fontsize',fontsize_label,'Fontweigh
t','bold','fontname','Times New Roman');
h = legend('Bv1=3 ', 'Bv1=6 ', 'Bv1=9');
set(h,'fontsize',fontsize_legend,'location','Best')

hfig = figure(1);
```

```
figWidth = 7.99;
figHeight = 5.5;
set(hfig,'PaperUnits','centimeters');
set(hfig,'PaperPosition',[0 0 figWidth figHeight]);
fileout = 'figure 8(a)';
print(hfig,[fileout],'-r500','-dtiff');

clc;clear;
linewidth_line = 0.5;
linewidth_gca = 0.8;
fontsize_gca = 6;
fontsize_label = 8;
fontsize_legend = 4;
DeltaG=5,Tg1=6,Tg2=5,Fv=3,A=6,Tp1=3,Fp=2,Bg=1,Dg=2,DeltaV=5,Dv1=1,Tp2
=3,Bv2=1,Dv2=2,alpha=0.5,beta=0.5,Mp1=2,Cp1=2.5,Cp2=5;
figure(2)
for Bv1=3;
    [t,y]=ode45(@(t,y)
huanjing(t,y,DeltaG,Tg1,Tg2,Fv,A,Tp1,Fp,Bg,Dg,DeltaV,Bv1,Dv1,Tp2,Bv2,
Dv2,alpha,beta,Mp1,Cp1,Cp2),[0 10],[0.5,0.5,0.5]);
    plot(t,y(:,2),'k-
','lineWidth',linewidth_line,'markerfacecolor','k');
    hold on
end
for Bv1=6;
    [t,y]=ode45(@(t,y)
huanjing(t,y,DeltaG,Tg1,Tg2,Fv,A,Tp1,Fp,Bg,Dg,DeltaV,Bv1,Dv1,Tp2,Bv2,
Dv2,alpha,beta,Mp1,Cp1,Cp2),[0 10],[0.5,0.5,0.5]);
    plot(t,y(:,2),'k--
','lineWidth',linewidth_line,'markerfacecolor','k');
    hold on
end
for Bv1=9;
    [t,y]=ode45(@(t,y)
huanjing(t,y,DeltaG,Tg1,Tg2,Fv,A,Tp1,Fp,Bg,Dg,DeltaV,Bv1,Dv1,Tp2,Bv2,
Dv2,alpha,beta,Mp1,Cp1,Cp2),[0 10],[0.5,0.5,0.5]);

plot(t,y(:,2),'k:','lineWidth',linewidth_line,'markerfacecolor','k');
    hold on
end
set(gca,'XTick',[0:1:10],'YTick',[0:0.1:1],'linewidth',linewidth_gca,
'fontsize',fontsize_gca)
axis([0 10 0 1])
xlabel('Time',
```

```
'interpreter','latex','fontsize',fontsize_label,'Fontweight','bold','
fontname','Times New Roman');
ylabel('The probability of strategy
selection','interpreter','latex','fontsize',fontsize_label,'Fontweigh
t','bold','fontname','Times New Roman');
h = legend('Bv1=3 ', 'Bv1=6 ', 'Bv1=9');
set(h,'fontsize',fontsize_legend,'location','Best')

hfig = figure(2);
figWidth = 7.99;
figHeight = 5.5;
set(hfig,'PaperUnits','centimeters');
set(hfig,'PaperPosition',[0 0 figWidth figHeight]);
fileout = 'figure 8(b)';
print(hfig,[fileout],'-r500','-dtiff');

clc;clear;
linewidth_line = 0.5;
linewidth_gca = 0.8;
fontsize_gca = 6;
fontsize_label = 8;
fontsize_legend = 4;
DeltaG=5,Tg1=6,Tg2=5,Fv=3,A=6,Tp1=3,Fp=2,Bg=1,Dg=2,DeltaV=5,Dv1=1,Tp2
=3,Bv2=1,Dv2=2,alpha=0.5,beta=0.5,Mp1=2,Cp1=2.5,Cp2=5;
figure(3)
for Bv1=3;
    [t,y]=ode45(@(t,y)
huanjing(t,y,DeltaG,Tg1,Tg2,Fv,A,Tp1,Fp,Bg,Dg,DeltaV,Bv1,Dv1,Tp2,Bv2,
Dv2,alpha,beta,Mp1,Cp1,Cp2),[0 10],[0.5,0.5,0.5]);
    plot(t,y(:,3),'k-
','lineWidth',linewidth_line,'markerfacecolor','k');
    hold on
end
for Bv1=6;
    [t,y]=ode45(@(t,y)
huanjing(t,y,DeltaG,Tg1,Tg2,Fv,A,Tp1,Fp,Bg,Dg,DeltaV,Bv1,Dv1,Tp2,Bv2,
Dv2,alpha,beta,Mp1,Cp1,Cp2),[0 10],[0.5,0.5,0.5]);
    plot(t,y(:,3),'k--
','lineWidth',linewidth_line,'markerfacecolor','k');
    hold on
end
for Bv1=9;
    [t,y]=ode45(@(t,y)
huanjing(t,y,DeltaG,Tg1,Tg2,Fv,A,Tp1,Fp,Bg,Dg,DeltaV,Bv1,Dv1,Tp2,Bv2,
```

```
Dv2,alpha,beta,Mp1,Cp1,Cp2),[0 10],[0.5,0.5,0.5]);

plot(t,y(:,1),'k:','lineWidth',linewidth_line,'markerfacecolor','k');
    hold on
end
set(gca,'XTick',[0:1:10],'YTick',[0:0.1:1],'linewidth',linewidth_gca,
'fontsize',fontsize_gca)
axis([0 10 0 1])
xlabel('Time',
'interpreter','latex','fontsize',fontsize_label,'Fontweight','bold','
fontname','Times New Roman');
ylabel('The probability of strategy
selection','interpreter','latex','fontsize',fontsize_label,'Fontweigh
t','bold','fontname','Times New Roman');
h = legend('Bv1=3 ', 'Bv1=6 ', 'Bv1=9');
set(h,'fontsize',fontsize_legend,'location','Best')

hfig = figure(3);
figWidth = 7.99;
figHeight = 5.5;
set(hfig,'PaperUnits','centimeters');
set(hfig,'PaperPosition',[0 0 figWidth figHeight]);
fileout = 'figure 8(c)';
print(hfig,[fileout],'-r500','-dtiff');
```

Fig 6

```
clc;clear;
linewidth_line = 0.5;
linewidth_gca = 0.8;
fontsize_gca = 6;
fontsize_label = 8;
fontsize_legend = 4;
DeltaG=5,Tg1=6,Tg2=5,Fv=3,A=6,Tp1=3,Fp=2,Bg=1,Dg=2,DeltaV=5,Bv1=3,Dv1
=1,Tp2=3,Bv2=1,Dv2=2,beta=0.5,Mp1=2,Cp1=2.5,Cp2=5;
figure(1)
for alpha=0.3;
    [t,y]=ode45(@(t,y)
huanjing(t,y,DeltaG,Tg1,Tg2,Fv,A,Tp1,Fp,Bg,Dg,DeltaV,Bv1,Dv1,Tp2,Bv2,
Dv2,alpha,beta,Mp1,Cp1,Cp2),[0 10],[0.5,0.5,0.5]);
    plot(t,y(:,1),'k-
','lineWidth',linewidth_line,'markerfacecolor','k');
    hold on
end
for alpha=0.5;
```

```
    [t,y]=ode45(@(t,y)
huanjing(t,y,DeltaG,Tg1,Tg2,Fv,A,Tp1,Fp,Bg,Dg,DeltaV,Bv1,Dv1,Tp2,Bv2,
Dv2,alpha,beta,Mp1,Cp1,Cp2),[0 10],[0.5,0.5,0.5]);
    plot(t,y(:,1),'k--
','lineWidth',linewidth_line,'markerfacecolor','k');
    hold on
end
for alpha=0.7;
    [t,y]=ode45(@(t,y)
huanjing(t,y,DeltaG,Tg1,Tg2,Fv,A,Tp1,Fp,Bg,Dg,DeltaV,Bv1,Dv1,Tp2,Bv2,
Dv2,alpha,beta,Mp1,Cp1,Cp2),[0 10],[0.5,0.5,0.5]);

plot(t,y(:,1),'k:','lineWidth',linewidth_line,'markerfacecolor','k');
    hold on
end
set(gca,'XTick',[0:1:10],'YTick',[0:0.1:1],'linewidth',linewidth_gca,
'fontsize',fontsize_gca)
axis([0 10 0 1])
xlabel('Time',
'interpreter','latex','fontsize',fontsize_label,'Fontweight','bold','
fontname','Times New Roman');
ylabel('The probability of strategy
selection','interpreter','latex','fontsize',fontsize_label,'Fontweigh
t','bold','fontname','Times New Roman');
h = legend('alpha=0.3', 'alpha=0.5 ', 'alpha=0.7');
set(h,'fontsize',fontsize_legend,'location','Best')

hfig = figure(1);
figWidth = 7.99;
figHeight = 5.5;
set(hfig,'PaperUnits','centimeters');
set(hfig,'PaperPosition',[0 0 figWidth figHeight]);
fileout = 'figure 9(a)';
print(hfig,[fileout],'-r500','-dtiff');

clc;clear;
linewidth_line = 0.5;
linewidth_gca = 0.8;
fontsize_gca = 6;
fontsize_label = 8;
fontsize_legend = 4;
DeltaG=5,Tg1=6,Tg2=5,Fv=3,A=6,Tp1=3,Fp=2,Bg=1,Dg=2,DeltaV=5,Bv1=3,Dv1
=1,Tp2=3,Bv2=1,Dv2=2,beta=0.5,Mp1=2,Cp1=2.5,Cp2=5;
figure(2)
```

```
for alpha=0.3;
    [t,y]=ode45(@(t,y)
huanjing(t,y,DeltaG,Tg1,Tg2,Fv,A,Tp1,Fp,Bg,Dg,DeltaV,Bv1,Dv1,Tp2,Bv2,
Dv2,alpha,beta,Mp1,Cp1,Cp2),[0 10],[0.5,0.5,0.5]);
    plot(t,y(:,2),'k-
','lineWidth',linewidth_line,'markerfacecolor','k');
    hold on
end
for alpha=0.5;
    [t,y]=ode45(@(t,y)
huanjing(t,y,DeltaG,Tg1,Tg2,Fv,A,Tp1,Fp,Bg,Dg,DeltaV,Bv1,Dv1,Tp2,Bv2,
Dv2,alpha,beta,Mp1,Cp1,Cp2),[0 10],[0.5,0.5,0.5]);
    plot(t,y(:,2),'k--
','lineWidth',linewidth_line,'markerfacecolor','k');
    hold on
end
for alpha=0.7;
    [t,y]=ode45(@(t,y)
huanjing(t,y,DeltaG,Tg1,Tg2,Fv,A,Tp1,Fp,Bg,Dg,DeltaV,Bv1,Dv1,Tp2,Bv2,
Dv2,alpha,beta,Mp1,Cp1,Cp2),[0 10],[0.5,0.5,0.5]);

plot(t,y(:,2),'k:','lineWidth',linewidth_line,'markerfacecolor','k');
    hold on
end
set(gca,'XTick',[0:1:10],'YTick',[0:0.1:1],'linewidth',linewidth_gca,
'fontsize',fontsize_gca)
axis([0 10 0 1])
xlabel('Time',
'interpreter','latex','fontsize',fontsize_label,'Fontweight','bold','
fontname','Times New Roman');
ylabel('The probability of strategy
selection','interpreter','latex','fontsize',fontsize_label,'Fontweigh
t','bold','fontname','Times New Roman');
h = legend('alpha=0.3', 'alpha=0.5 ', 'alpha=0.7');
set(h,'fontsize',fontsize_legend,'location','Best')

hfig = figure(2);
figWidth = 7.99;
figHeight = 5.5;
set(hfig,'PaperUnits','centimeters');
set(hfig,'PaperPosition',[0 0 figWidth figHeight]);
fileout = 'figure 9(b)';
print(hfig,[fileout],'-r500','-dtiff');
```

```
clc;clear;
linewidth_line = 0.5;
linewidth_gca = 0.8;
fontsize_gca = 6;
fontsize_label = 8;
fontsize_legend = 4;
DeltaG=5,Tg1=6,Tg2=5,Fv=3,A=6,Tp1=3,Fp=2,Bg=1,Dg=2,DeltaV=5,Bv1=3,Dv1
=1,Tp2=3,Bv2=1,Dv2=2,beta=0.5,Mp1=2,Cp1=2.5,Cp2=5;
figure(3)
for alpha=0.3;
    [t,y]=ode45(@(t,y)
huanjing(t,y,DeltaG,Tg1,Tg2,Fv,A,Tp1,Fp,Bg,Dg,DeltaV,Bv1,Dv1,Tp2,Bv2,
Dv2,alpha,beta,Mp1,Cp1,Cp2),[0 10],[0.5,0.5,0.5]);
    plot(t,y(:,3),'k-
','lineWidth',linewidth_line,'markerfacecolor','k');
    hold on
end
for alpha=0.5;
    [t,y]=ode45(@(t,y)
huanjing(t,y,DeltaG,Tg1,Tg2,Fv,A,Tp1,Fp,Bg,Dg,DeltaV,Bv1,Dv1,Tp2,Bv2,
Dv2,alpha,beta,Mp1,Cp1,Cp2),[0 10],[0.5,0.5,0.5]);
    plot(t,y(:,3),'k--
','lineWidth',linewidth_line,'markerfacecolor','k');
    hold on
end
for alpha=0.7;
    [t,y]=ode45(@(t,y)
huanjing(t,y,DeltaG,Tg1,Tg2,Fv,A,Tp1,Fp,Bg,Dg,DeltaV,Bv1,Dv1,Tp2,Bv2,
Dv2,alpha,beta,Mp1,Cp1,Cp2),[0 10],[0.5,0.5,0.5]);

plot(t,y(:,3),'k:','lineWidth',linewidth_line,'markerfacecolor','k');
    hold on
end
set(gca,'XTick',[0:1:10],'YTick',[0:0.1:1],'linewidth',linewidth_gca,
'fontsize',fontsize_gca)
axis([0 10 0 1])
xlabel('Time',
'interpreter','latex','fontsize',fontsize_label,'Fontweight','bold','
fontname','Times New Roman');
ylabel('The probability of strategy
selection','interpreter','latex','fontsize',fontsize_label,'Fontweigh
t','bold','fontname','Times New Roman');
h = legend('alpha=0.3', 'alpha=0.5 ', 'alpha=0.7');
set(h,'fontsize',fontsize_legend,'location','Best')
```

```
hfig = figure(3);
figWidth = 7.99;
figHeight = 5.5;
set(hfig,'PaperUnits','centimeters');
set(hfig,'PaperPosition',[0 0 figWidth figHeight]);
fileout = 'figure 9(c)';
print(hfig,[fileout],'-r500','-dtiff');
```

Fig 7

```
clc;clear;
linewidth_line = 0.5;
linewidth_gca = 0.8;
fontsize_gca = 6;
fontsize_label = 8;
fontsize_legend = 4;
DeltaG=5,Tg1=6,Tg2=5,Fv=3,A=6,Tp1=3,Fp=2,Bg=1,DeltaV=5,Bv1=3,Dv1=1,Tp
2=3,Bv2=1,alpha=0.5,beta=0.5,Mp1=2,Cp1=2.5,Cp2=5;
figure(1)
for Bg=1,Bv2=1,Dg=1,Dv2=1;
    [t,y]=ode45(@(t,y)
huanjing(t,y,DeltaG,Tg1,Tg2,Fv,A,Tp1,Fp,Bg,Dg,DeltaV,Bv1,Dv1,Tp2,Bv2,
Dv2,alpha,beta,Mp1,Cp1,Cp2),[0 10],[0.5,0.5,0.5]);
    plot(t,y(:,1),'k-
','lineWidth',linewidth_line,'markerfacecolor','k');
    hold on
end
for Bg=1,Bv2=1,Dg=4,Dv2=4;
    [t,y]=ode45(@(t,y)
huanjing(t,y,DeltaG,Tg1,Tg2,Fv,A,Tp1,Fp,Bg,Dg,DeltaV,Bv1,Dv1,Tp2,Bv2,
Dv2,alpha,beta,Mp1,Cp1,Cp2),[0 10],[0.5,0.5,0.5]);
    plot(t,y(:,1),'k--
','lineWidth',linewidth_line,'markerfacecolor','k');
    hold on
end
for Bg=4,Bv2=4,Dg=1,Dv2=1;
    [t,y]=ode45(@(t,y)
huanjing(t,y,DeltaG,Tg1,Tg2,Fv,A,Tp1,Fp,Bg,Dg,DeltaV,Bv1,Dv1,Tp2,Bv2,
Dv2,alpha,beta,Mp1,Cp1,Cp2),[0 10],[0.5,0.5,0.5]);

plot(t,y(:,1),'k:','lineWidth',linewidth_line,'markerfacecolor','k');
    hold on
end
set(gca,'XTick',[0:1:10],'YTick',[0:0.1:1],'linewidth',linewidth_gca,
```

```
'fontsize',fontsize_gca)
axis([0 10 0 1])
xlabel('Time',
'interpreter','latex','fontsize',fontsize_label,'Fontweight','bold','
fontname','Times New Roman');
ylabel('The probability of strategy
selection','interpreter','latex','fontsize',fontsize_label,'Fontweigh
t','bold','fontname','Times New Roman');
h = legend('Bg=1,Bv2=1,Dg=1,Dv2=1', 'Bg=1,Bv2=1,Dg=4,Dv2=4',
'Bg=4,Bv2=4,Dg=1,Dv2=1');
set(h,'fontsize',fontsize_legend,'location','Best')

hfig = figure(1);
figWidth = 7.99;
figHeight = 5.5;
set(hfig,'PaperUnits','centimeters');
set(hfig,'PaperPosition',[0 0 figWidth figHeight]);
fileout = 'figure 10(a)';
print(hfig,[fileout],'-r500','-dtiff');

clc;clear;
linewidth_line = 0.5;
linewidth_gca = 0.8;
fontsize_gca = 6;
fontsize_label = 8;
fontsize_legend = 4;
DeltaG=5,Tg1=6,Tg2=5,Fv=3,A=6,Tp1=3,Fp=2,Bg=1,DeltaV=5,Bv1=3,Dv1=1,Tp
2=3,Bv2=1,alpha=0.5,beta=0.5,Mp1=2,Cp1=2.5,Cp2=5;
figure(2)
for Bg=1,Bv2=1,Dg=1,Dv2=1;
    [t,y]=ode45(@(t,y)
huanjing(t,y,DeltaG,Tg1,Tg2,Fv,A,Tp1,Fp,Bg,Dg,DeltaV,Bv1,Dv1,Tp2,Bv2,
Dv2,alpha,beta,Mp1,Cp1,Cp2),[0 10],[0.5,0.5,0.5]);
    plot(t,y(:,2),'k-
','lineWidth',linewidth_line,'markerfacecolor','k');
    hold on
end
for Bg=1,Bv2=1,Dg=4,Dv2=4;
    [t,y]=ode45(@(t,y)
huanjing(t,y,DeltaG,Tg1,Tg2,Fv,A,Tp1,Fp,Bg,Dg,DeltaV,Bv1,Dv1,Tp2,Bv2,
Dv2,alpha,beta,Mp1,Cp1,Cp2),[0 10],[0.5,0.5,0.5]);
    plot(t,y(:,2),'k--
','lineWidth',linewidth_line,'markerfacecolor','k');
    hold on
```

```
end
for Bg=4,Bv2=4,Dg=1,Dv2=1;
    [t,y]=ode45(@(t,y)
huanjing(t,y,DeltaG,Tg1,Tg2,Fv,A,Tp1,Fp,Bg,Dg,DeltaV,Bv1,Dv1,Tp2,Bv2,
Dv2,alpha,beta,Mp1,Cp1,Cp2),[0 10],[0.5,0.5,0.5]);

plot(t,y(:,2),'k:','lineWidth',linewidth_line,'markerfacecolor','k');
    hold on
end
set(gca,'XTick',[0:1:10],'YTick',[0:0.1:1],'linewidth',linewidth_gca,
'fontsize',fontsize_gca)
axis([0 10 0 1])
xlabel('Time',
'interpreter','latex','fontsize',fontsize_label,'Fontweight','bold','
fontname','Times New Roman');
ylabel('The probability of strategy
selection','interpreter','latex','fontsize',fontsize_label,'Fontweigh
t','bold','fontname','Times New Roman');
h = legend('Bg=1,Bv2=1,Dg=1,Dv2=1', 'Bg=1,Bv2=1,Dg=4,Dv2=4',
'Bg=4,Bv2=4,Dg=1,Dv2=1');
set(h,'fontsize',fontsize_legend,'location','Best')

hfig = figure(2);
figWidth = 7.99;
figHeight = 5.5;
set(hfig,'PaperUnits','centimeters');
set(hfig,'PaperPosition',[0 0 figWidth figHeight]);
fileout = 'figure 10(b)';
print(hfig,[fileout],'-r500','-dtiff');

clc;clear;
linewidth_line = 0.5;
linewidth_gca = 0.8;
fontsize_gca = 6;
fontsize_label = 8;
fontsize_legend = 4;
DeltaG=5,Tg1=6,Tg2=5,Fv=3,A=6,Tp1=3,Fp=2,Bg=1,DeltaV=5,Bv1=3,Dv1=1,Tp
2=3,Bv2=1,alpha=0.5,beta=0.5,Mp1=2,Cp1=2.5,Cp2=5;
figure(3)
for Bg=1,Bv2=1,Dg=1,Dv2=1;
    [t,y]=ode45(@(t,y)
huanjing(t,y,DeltaG,Tg1,Tg2,Fv,A,Tp1,Fp,Bg,Dg,DeltaV,Bv1,Dv1,Tp2,Bv2,
Dv2,alpha,beta,Mp1,Cp1,Cp2),[0 10],[0.5,0.5,0.5]);
    plot(t,y(:,3),'k-
```

```
','lineWidth',linewidth_line,'markerfacecolor','k');
    hold on
end
for Bg=1,Bv2=1,Dg=4,Dv2=4;
    [t,y]=ode45(@(t,y)
huanjing(t,y,DeltaG,Tg1,Tg2,Fv,A,Tp1,Fp,Bg,Dg,DeltaV,Bv1,Dv1,Tp2,Bv2,
Dv2,alpha,beta,Mp1,Cp1,Cp2),[0 10],[0.5,0.5,0.5]);
    plot(t,y(:,3),'k--
','lineWidth',linewidth_line,'markerfacecolor','k');
    hold on
end
for Bg=4,Bv2=4,Dg=1,Dv2=1;
    [t,y]=ode45(@(t,y)
huanjing(t,y,DeltaG,Tg1,Tg2,Fv,A,Tp1,Fp,Bg,Dg,DeltaV,Bv1,Dv1,Tp2,Bv2,
Dv2,alpha,beta,Mp1,Cp1,Cp2),[0 10],[0.5,0.5,0.5]);

plot(t,y(:,3),'k:','lineWidth',linewidth_line,'markerfacecolor','k');
    hold on
end
set(gca,'XTick',[0:1:10],'YTick',[0:0.1:1],'linewidth',linewidth_gca,
'fontsize',fontsize_gca)
axis([0 10 0 1])
xlabel('Time',
'interpreter','latex','fontsize',fontsize_label,'Fontweight','bold','
fontname','Times New Roman');
ylabel('The probability of strategy
selection','interpreter','latex','fontsize',fontsize_label,'Fontweigh
t','bold','fontname','Times New Roman');
h = legend('Bg=1,Bv2=1,Dg=1,Dv2=1', 'Bg=1,Bv2=1,Dg=4,Dv2=4',
'Bg=4,Bv2=4,Dg=1,Dv2=1');
set(h,'fontsize',fontsize_legend,'location','Best')

hfig = figure(3);
figWidth = 7.99;
figHeight = 5.5;
set(hfig,'PaperUnits','centimeters');
set(hfig,'PaperPosition',[0 0 figWidth figHeight]);
fileout = 'figure 10(c)';
print(hfig,[fileout],'-r500','-dtiff');
```
